# Integrated Multi-Omics Analysis Reveals Lipid Metabolism-Mediated Preservation of Postharvest Broccoli Yellowing by Static Magnetic Field

**DOI:** 10.3390/plants15060870

**Published:** 2026-03-11

**Authors:** Yi-Bin Lu, Jin-Feng Huang, Xu-Feng Chen, Wei-Lin Huang, Li-Song Chen

**Affiliations:** 1 School of Food Engineering, Zhangzhou Institute of Technology, Zhangzhou 363000, China; huangjinfeng@fjzzit.edu.cn; 2 College of Resources and Environment, Fujian Agriculture and Forestry University, Fuzhou 350002, China; chengxufeng1996@163.com; 3 College of Environmental and Biological Engineering, Putian University, Putian 351100, China

**Keywords:** *Brassica oleracea* L. var. italica, lysophospholipid, postharvest yellowing, senescence, sphingolipid

## Abstract

Broccoli (*Brassica oleracea* L. var. italica) is prone to rapid yellowing when stored at ambient temperature after harvest due to membrane damage. Here, freshly harvested broccoli florets were stored in a static magnetic field (5 mT) at 20 °C. The current results demonstrated that a static magnetic field lowered postharvest yellowing (chlorophyll breakdown), water loss, and oxidative stress. An integrated transcriptome and metabolome analysis suggested that static magnetic field-mediated alleviation of postharvest yellowing and senescence of broccoli florets involved the following factors: (1) downregulating the expression of genes related to organ senescence; (2) delaying the breakdown of chlorophylls through preventing the upregulation of chlorophyll degradation-related genes and the increase in oxidative stress; (3) alleviating cellular energy imbalance by upregulated fatty acid oxidation (as indicated by decreased free fatty acids) to reduce water loss and oxidative stress and to maintain membrane integrity; (4) increasing the abundances of lysophospholipids and sphingolipids and preventing the decrease in phosphatidylcholine abundance to lower water loss and oxidative stress, inhibit ethylene production, delay chlorophyll degradation, and keep membrane integrity; (5) reducing water loss via increasing cutin, suberin, and wax biosynthesis and stomatal closure brought about by upregulated expression of phospholipase D genes; and (6) preventing the increase in malondialdehyde (MDA) content, electrolyte leakage, and weight loss rate. To conclude, this work provided some novel data elucidating the underlying mechanism by which a static magnetic field delayed postharvest yellowing and senescence of broccoli florets. A static magnetic field could retard postharvest deterioration of broccoli florets, thereby providing a clean and non-thermal method for their green preservation.

## 1. Introduction

Broccoli (*Brassica oleracea* L. var. italica) is a widely consumed vegetable recognized for its rich nutritional content and health benefits. It is a significant source of vitamins, minerals, and dietary fiber as well as various bioactive compounds, including phenolics, flavonoids, and glucosinolates, which are known for their anticancer properties [1]. However, postharvest broccoli is prone to quality deterioration due to its vigorous respiration and active metabolism, leading to rapid yellowing, which significantly affects its visual appeal and nutritional value, leading to economic losses and waste [2].

In an effort to extend the limited commercial shelf-life, various physical [3,4,5,6], chemical [7,8,9], and biological [10,11,12] preservation techniques have been explored to maintain postharvest quality of broccoli. Physical treatments including storage temperature treatment [3], modified atmosphere packaging [4], UV treatment [5], LED lighting [6] and so on have gained attention due to being residue free. Chemical treatments like 1-methylcyclopropene (1-MCP) [7], diacetyl [8], and H_2_O_2_ [9] are effective and low cost but often criticized for potential chemical residues and safety concerns. Biological preservation methods, such as plant hormones [10], folic acid [11], amino acids (AAs) [12], and melatonin [2], can effectively delay the yellowing and senescence of postharvest broccoli but may suffer from inconsistent efficacy and higher production costs. Thus, physical preservation methods have emerged as the preferred choice for broccoli postharvest preservation.

The storability of harvested vegetables and fruit is associated with the integrity of cell membrane structure, which ensures the functionality of cell and disease resistance in fruit and vegetables [13,14]. Methyl jasmonate (MeJA) can protect the cell membrane integrity of bananas and significantly delay ripening and senescence [15]. The integrity of cell membranes is fundamentally linked to lipid metabolism [13], including fatty acid synthesis/degradation, fatty acid β-oxidation, membrane lipid remodeling, antioxidant defense, and barrier formation. The disruptions in lipid metabolic pathways increases the permeability of the cell membrane and lead to fruit senescence. A membrane lipidomic approach revealed that the pitting of blueberries caused by cold stress during refrigeration was driven by membrane lipid remodeling and peroxidation [16]. Membrane lipid degradation and peroxidation also resulted in membrane damage and accelerated chilling injury of bananas [17]. Exogenous H_2_O_2_ triggered lipoxygenase (LOX)-mediated membrane lipid peroxidation, impaired membrane integrity, and consequently accelerated pericarp browning in longan [18]. Postharvest sodium nitroprusside (SNP) treatment was reported to maintain blueberry quality by enhancing fatty acid synthesis and suppressing membrane lipid peroxidation [19]. These studies indicated that membrane lipid metabolism played a key role in preserving cell membrane structural integrity and delaying fruit and vegetable senescence in harvested fruit and vegetables.

In recent years, the magnetic field (MF) has emerged as a promising non-thermal physical preservation technology characterized by energy efficiency, cost-effectiveness, environmental friendliness, and operational simplicity [20,21]. It effectively extends the shelf life and maintains the quality of various fruit and vegetables by suppressing respiration rates, regulating enzyme activities, reducing oxidative stress, maintaining cellular structure, and inhibiting microbial growth in diverse horticultural products [22,23,24,25,26,27,28]. It was reported that a MF could delay senescence and decay of fruit through modulating the membrane lipid bilayer in the cell membrane and maintaining membrane integrity and function [22,25,28]. Short-term high-intensity static MF (SMF) treatment could significantly suppress the respiration, energy-related pathways, and relative electrolytic leakage of fresh-cut young ginger [26]. The combined application of a pulsed MF and cold water shock could make cucumbers have better preservation quality via boosting catalase activity and improving cell membrane integrity [24]. Zhao et al. [23] revealed that the SMF could suppress the rise in malondialdehyde (MDA) content and reduce electrolyte leakage in cucumber during storage. This drove us to hypothesize that an SMF can delay the senescence of fruit and vegetables after harvest via maintaining membrane structure integrity and functions.

With the development of omics technologies, integrated transcriptomics and metabolomics have been applied as powerful tools to reveal the causes of quality deterioration during postharvest processing of fruits and vegetables as well as the underlying mechanisms of different preservation methods to delay the senescence of fruit and vegetables after harvest. Such data have been reported in various fruit and vegetables such as strawberry [29], broccoli [30], Zizania latifolia [31], grape [32], apple [33], kiwifruit [34], blueberry [35], goji berry [36], yam [37] and so on.

So far, there has been no systematic evaluation of the effect of an SMF on broccoli preservation, nor have omics methods been used to elucidate the underlying mechanisms by which an SMF delays broccoli senescence. The present study is devoted to investigating the effect of an SMF on delaying postharvest yellowing and senescence of broccoli. It is speculated that (1) an SMF delays the deterioration of postharvest broccoli by comprehensively regulating key physiological, molecular, and metabolic processes, and (2) the intrinsic molecular mechanisms of an SMF delaying broccoli senescence might be related to lipid metabolism and maintenance of membrane integrity. To verify this hypothesis, a series of analyses including gene expression, metabolic profiles, and associated physiological parameters were performed on postharvest broccoli florets stored with or without SMF treatment so as to elucidate the underlying mechanisms.

## 2. Results

### 2.1. Changes in Visual Quality

**Figure 1:**
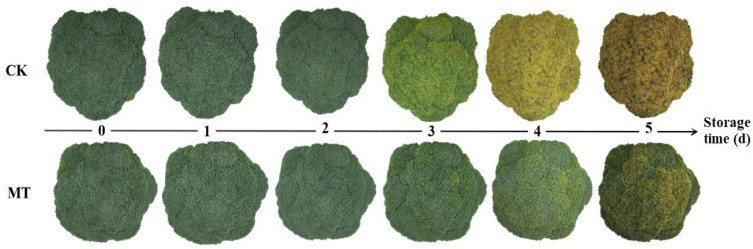
Visual quality of representative broccoli heads during storage at 20 ± 0.5 °C and 0 mT (CK) or 5 mT (MT) static magnetic field.

Compared with the control, SMF treatment (MT) could delay the yellowing of broccoli florets. Static magnetic field-treated broccoli maintained their green color after 3 d of storage, while the CK had already turned yellow after 3 d of storage. After being stored for 4 to 5 d, the broccoli heads treated with MT remained greener than the control (Figure 1).

**Figure 2:**
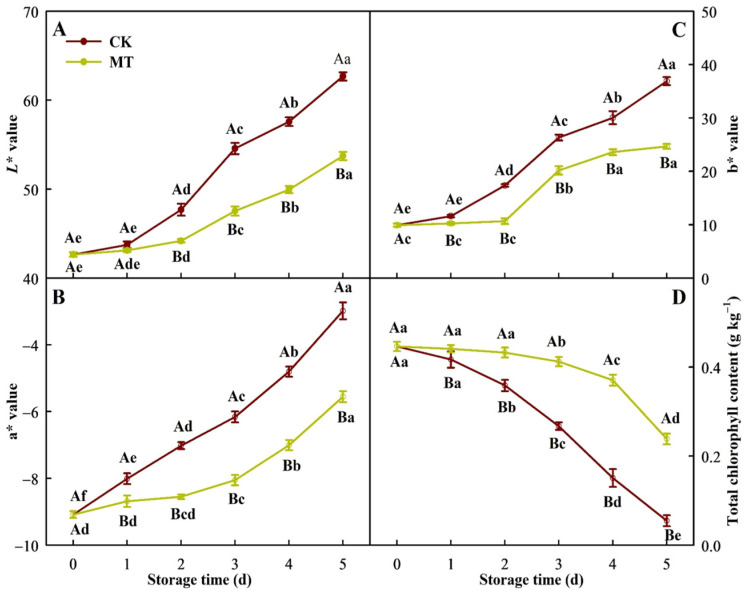
L* (**A**), a* (**B**), b* (**C**) and total chlorophyll content expressed on a fresh weight basis (**D**) of broccoli florets during storage at 20 ± 0.5 °C and 0 mT (CK) or 5 mT (MT) static magnetic field. Data were the mean ± SD (*n* = 3). Different capital letters denote statistically significant differences between samples subjected to different treatments on the same day (*p* < 0.05), while different lowercase letters indicate statistically significant differences across storage times within the same treatment (*p* < 0.05).

The color of broccoli is a key indicator of its yellowing degree. The research showed broccoli florets gradually turned from green to yellow, accompanied by an increase in L*, a*, and b* and a decrease in chlorophyll content, with a slower change in broccoli florets treated with 5 mT SMF than with 0 mT SMF (Figure 2), implying that 5 mT SMF delayed the yellowing of postharvest broccoli florets.

### 2.2. Effects of SMF on MDA Content, Electrolyte Leakage, and Weight Loss Rate in Broccoli Florets

**Figure 3:**
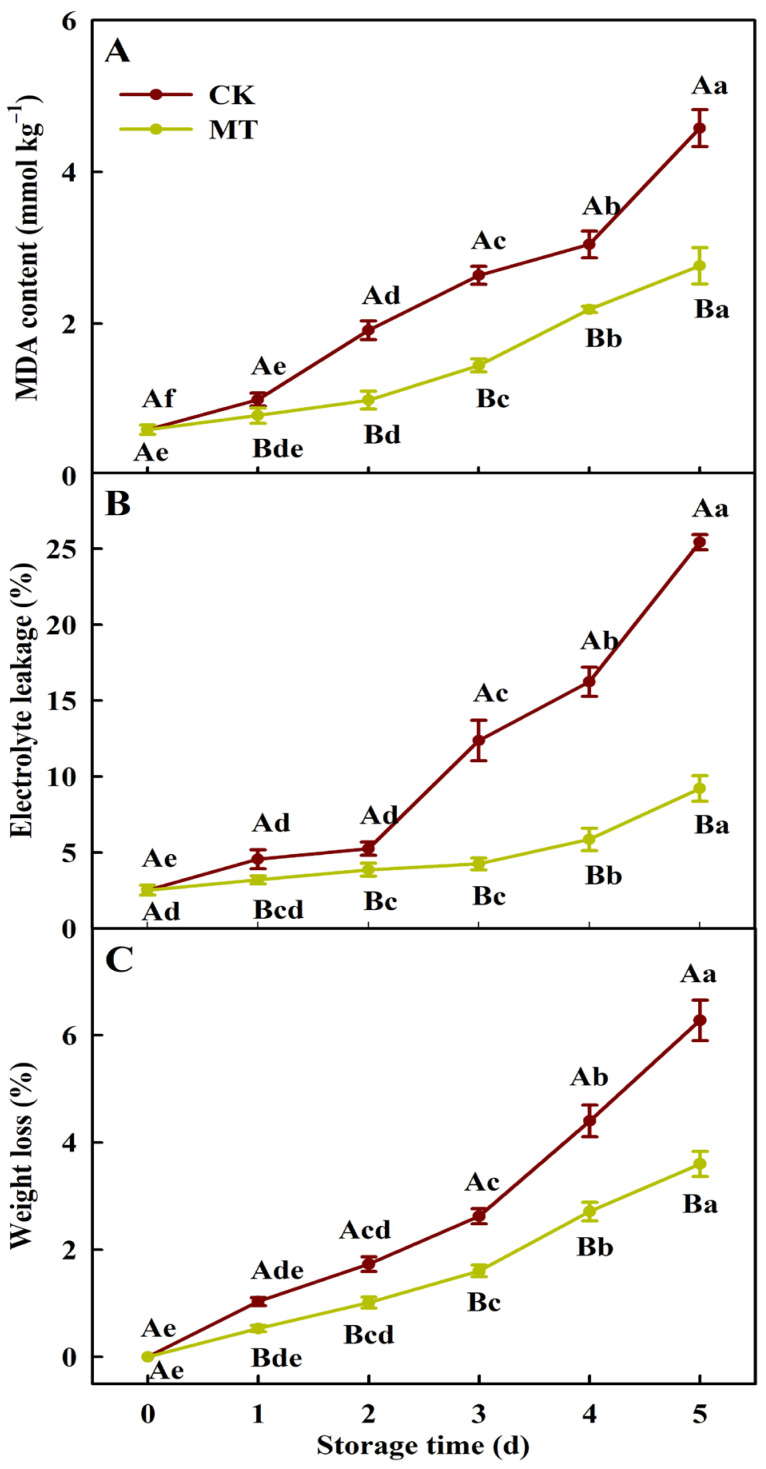
Malondialdehyde content expressed on a fresh weight basis (**A**), electrolyte leakage (**B**) and weight loss rate (**C**) of broccoli florets during storage at 20 ± 0.5 °C and 0 mT (CK) or 5 mT (MT) static magnetic field. Data were the mean ± SD (*n* = 3). Different capital letters denote statistically significant differences between samples subjected to different treatments on the same day (*p* < 0.05), while different lowercase letters indicate statistically significant differences across storage times within the same treatment (*p* < 0.05).

The research showed that MDA content (Figure 3A), electrolyte leakage (Figure 3B), and weight loss rate (Figure 3C) in broccoli florets continuously increased over storage time, with a greater increase in broccoli florets treated with 0 mT SMF than with 5 mT SMF. Malondialdehyde content and electrolyte leakage increased from 0.60 mmol kg^−1^ and 2.5% to 4.58 mmol kg^−1^ and 25.5% (2.86 mmol kg^−1^ and 9.2%) in broccoli florets treated with 0 mT (5 mT) SMF, respectively. By the fifth day of storage, the weight loss rates were 6.3% and 3.6% in broccoli florets treated with 0 mT and 5 mT SMF, respectively. To conclude, 5 mT SMF prevented the increase in MDA content, electrolyte leakage, and weight loss rate in broccoli florets during the storage period.

### 2.3. Pearson’s Correlation Coefficient Matrix for the Mean Values of MDA Content, Electrolyte Leakage, Weight Loss Rate, L*, a*, b*, and Chlorophyll Content

**Figure 4:**
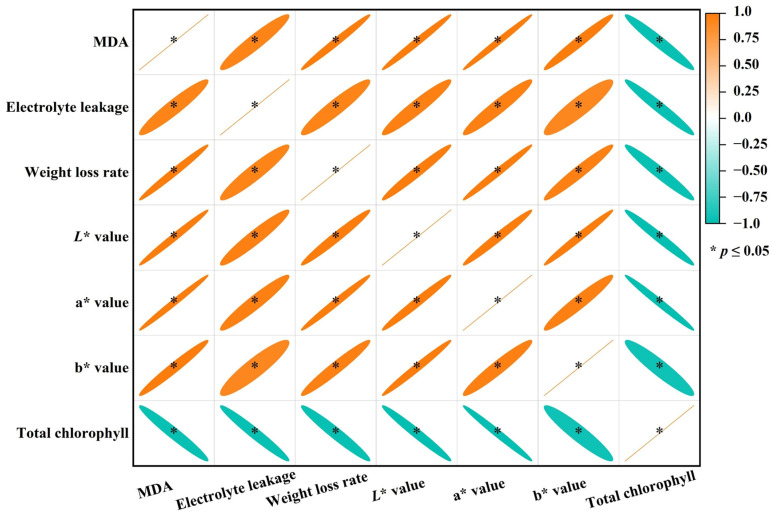
Pearson’s correlation coefficient matrix for the mean values of seven indexes.

The regression analysis revealed a significant positive correlation between any two parameters of MDA content, electrolyte leakage, weight loss rate, L*, a*, and b*, and that total chlorophyll content was significantly negatively related to the MDA content, electrolyte leakage, weight loss rate, L*, a *, and b * (Figure 4).

### 2.4. Transcriptome Profiles in Broccoli Florets

**Figure 5:**
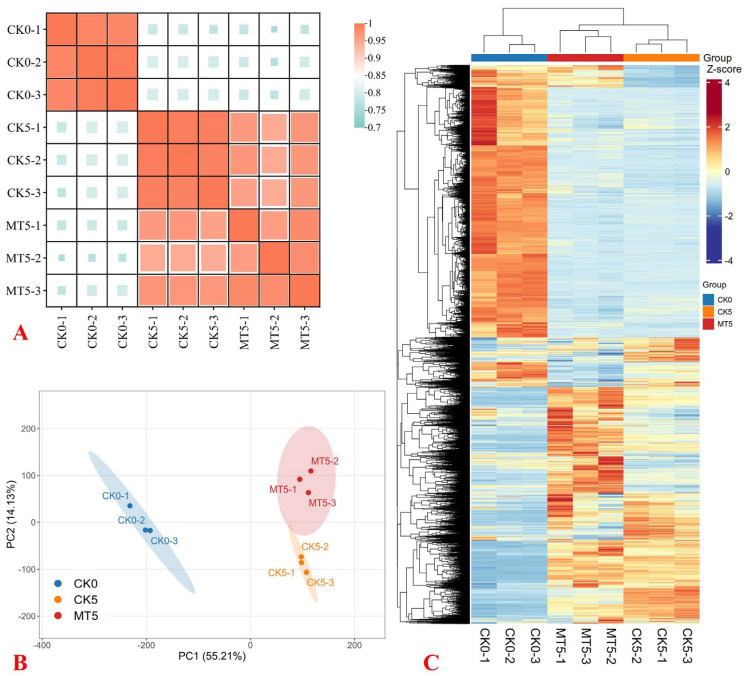
Pearson’s correlation coefficient (PCC) matrix (**A**) and principal component analysis (PCA) plot (**B**) of genes. Hierarchical cluster analysis (HCA) of DEGs (**C**) identified in CK5 vs. CK0, MT5 vs. CK0, and MT5 vs. CK5.

The high percentage of clean reads (98.09–98.68%), Q30 (90.29–94.03%) (Table S2), and Pearson’s correlation coefficient between biological replicates (Figure 5A) indicated that the RNA-Seq data were reliable. This was also supported by the PCA and the HCA (Figure 5B,C). The research showed that 77.66–86.44% and 2.35–2.68% of the clean reads were uniquely and multiply mapped to the broccoli reference genome, respectively (Table S3).

**Figure 6:**
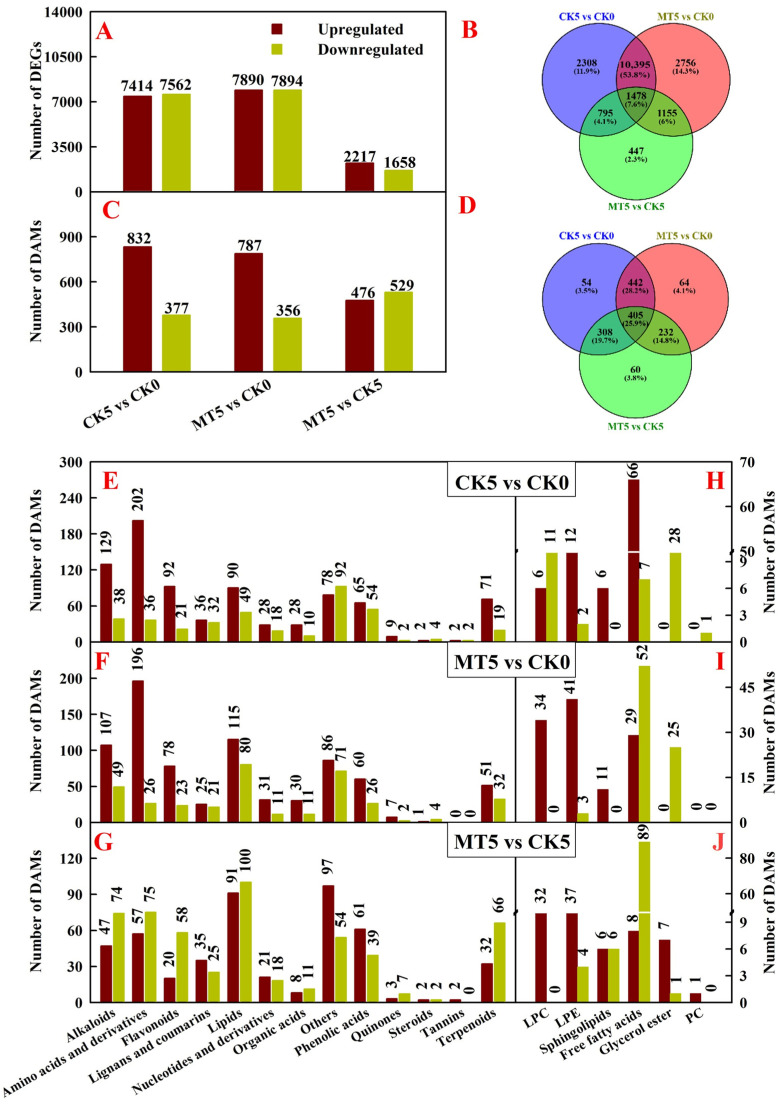
Upregulated and downregulated genes (**A**), Venn analysis of DEGs (**B**), upregulated and downregulated metabolites (**C**,**E**–**G**) and lipids (**H**–**J**), and Venn analysis of DAMs (**D**) detected in CK5 vs. CK0, MT5 vs. CK0, and MT5 vs. CK5. LPC, lysophosphatidylcholine; LPE, lysophosphatidylethanolamine; PC, phosphatidylcholine.

The research detected 7414, 7890, and 2217 upregulated genes and 7562, 7894, and 1658 downregulated genes in CK5 vs. CK0, MT5 vs. CK0, and MT5 vs. CK5, respectively (Figure 6A and Tables S4–S6). The research obtained a total of 19,334 DEGs in the three comparison groups, 2308, 2756, and 447 DEGs of which were identified only in CK5 vs. CK0, MT5 vs. CK0, and MT5 vs. CK5, respectively. Only 1478 DEGs were co-identified in the three comparison groups (Figure 6B).

In CK5 vs. CK0, 11,239, 10,238, and 10,293 DEGs were enriched to 524, 1144, and 3046 GO terms in cellular component (CC), molecular function (MF), and biological process (BP), respectively (Table S7). In MT5 vs. CK0, 11,865, 10,795, and 10,911 DEGs were enriched to 523, 1146, and 3055 GO terms in CC, MF, and BP, respectively (Table S8). In MT5 vs. CK5, 2936, 2679 and 2699 DEGs were enriched to 422, 976 and 2656 GO terms in CC, MF and BP, respectively (Table S9). It was observed that 4861, 5160, and 1360 DEGs were enriched to 142, 142, and 133 KEGG pathways in CK5 vs. CK0, MT5 vs. CK0, and MT5 vs. CK5, respectively (Tables S10–S12).

### 2.5. RT-qPCR Verification for Transcriptome Data

To verify the RNA-Seq data, 10 DEGs related to lipid metabolism were chosen for RT-qPCR analysis, namely LOX3 (LOC106312730), FACT (LOC106332210), C70B1 (LOC106299122), PLDZ1 (LOC106295624), BGAL6 (LOC106328575), GPAT6 (LOC106317133), DGAT1 (LOC106301685), KCS2 (LOC106295733), LACS1 (LOC106342148), and CER3 (LOC106327484). As shown in Figure S1, there is a significant positive correlation between RT-qPCR data and transcriptome data, demonstrating the credibility of the transcriptomic data (Figure S1 and Tables S4–S6).

### 2.6. Metabolome Profiles in Broccoli Florets

**Figure 7:**
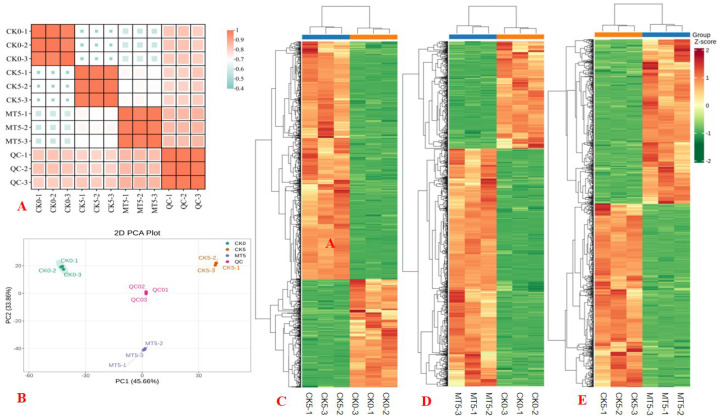
The Pearson’s correlation coefficient (PCC) matrix (**A**) and principal component analysis (PCA) plot (**B**) of genes. Hierarchical cluster analysis (HCA) of DAMs (**C**–**E**) identified in CK5 vs. CK0, MT5 vs. CK0, and MT5 vs. CK5.

The high PCCs suggested that the metabolomic data were reliable (Figure 7A). Principal component analysis showed that three biological repetitions of each treatment (mixed sample) were highly clustered together, and the three treatments CK0, CK5, and MT5 and the mixed sample were highly separated (Figure 7B), implying that the data are reliable, and SMF and storage time significantly affected the metabolite abundances of broccoli florets. Hierarchical cluster analysis revealed all biological replicates within the same treatment clustered together, with clear separation between the compared treatments (Figure 7C–E).

Orthogonal partial least squares discriminant analysis revealed distinct separation between treatment groups and high clustering of the three biological replicates within each group (Figure S2A–C). The Y2R and Q2 of OPLS-DA models were 1 (*p* < 0.05) and 0.996 (*p* < 0.05), 1 (*p* < 0.05) and 0.994 (*p* < 0.05), and 1 (*p* < 0.05) and 0.991 (*p* < 0.05) in CK5 vs. CK0, MT5 vs. CK0, and MT5 vs. CK5, respectively (Figure S2D–F). Therefore, these models were stable and reliable.

The research detected 832, 787, and 476 upregulated metabolites and 377, 356, and 529 downregulated metabolites in CK5 vs. CK0, MT5 vs. CK0, and MT5 vs. CK5, respectively. They mainly included lipids, alkaloids, AAs and derivatives, flavonoids, lignans and coumarins, organic acids (OAs), phenolic acids, and terpenoids (Tables S13–S16; Figure 6C,E–G). This study further analyzed lipids. The research detected 90 upregulated lipids in CK5 vs. CK0: 18 lysophospholipids (LPLs; six lysophosphatidylcholines (LPCs) + 12 lysophosphatidylethanolamines (LPEs), six sphingolipids, and 66 (22 saturated + 44 unsaturated) free fatty acids (FFAs). Additionally, there were 49 downregulated lipids in CK5 vs. CK0: 13 LPLs (two LPCs + 11 LPEs), one phospholipid (PL, phosphatidylcholine (PC)), seven (one saturated + six unsaturated) FFAs, and 28 glycerol esters. There were 115 upregulated lipids in MT5 vs. CK0: 75 LPLs (34 LPCs + 41 LPEs), 11 sphingolipids, and 29 (16 saturated + 13 unsaturated) FFAs. There were also 80 downregulated lipids in MT5 vs. CK0: three LPEs, 52 (eight saturated + 44 unsaturated) FFAs, and 25 glycerol esters. In MT5 vs. CK5, there were 91 upregulated lipids: 69 LPLs (32 LPCs + 37 LPEs), one PC, six sphingolipids, eight (three saturated + five unsaturated) FFAs, and seven glycerol esters. In MT5 vs. CK5, there were also 100 downregulated lipids: four LPEs, six sphingolipids, 89 (22 saturated + 67 unsaturated) FFAs, and one glycerol esters (Figure 6E–J and Table S17). To conclude, 5 mT SMF prevented storage-induced upregulation of FFAs and increased the accumulation of PLs during the storage period. The research identified a total of 1565 DAMs in the three comparison groups, 54, 64, and 60 DAMs of which were identified only in CK5 vs. CK0, MT5 vs. CK0, and MT5 vs. CK5, respectively. A total of 405 DAMs were co-identified in the three comparison groups (Figure 6D).

KEGG enrichment analysis showed that 226, 217, and 190 DAMs were enriched to 91, 86, and 92 KEGG pathways in CK5 vs. CK0, MT5 vs. CK0, and MT5 vs. CK5, respectively (Tables S18–S20).

### 2.7. Effects of SMF and Storage Time on the Expression Levels (Abundances) of Genes (Metabolites) Related to Senescence, Lipid Metabolism, and Chlorophyll Metabolism in Broccoli Florets

This study identified seven, six, and one upregulated and two, four, and three downregulated genes involved in “floral organ senescence” in CK5 vs. CK0, MT5 vs. CK0, and MT5 vs. CK5, respectively (Table 1). This implied that 5 mT SMF prevented the upregulation of floral organ senescence-related genes in broccoli florets during storage. Notably, the research identified more upregulated genes and fewer downregulated genes related to “plant organ senescence” and “leaf senescence” in MT5 vs. CK0 than in CK5 vs. CK0, as well as more upregulated than downregulated genes related to the two GO terms in MT5 vs. CK5 (Table 1).

**Figure 8:**
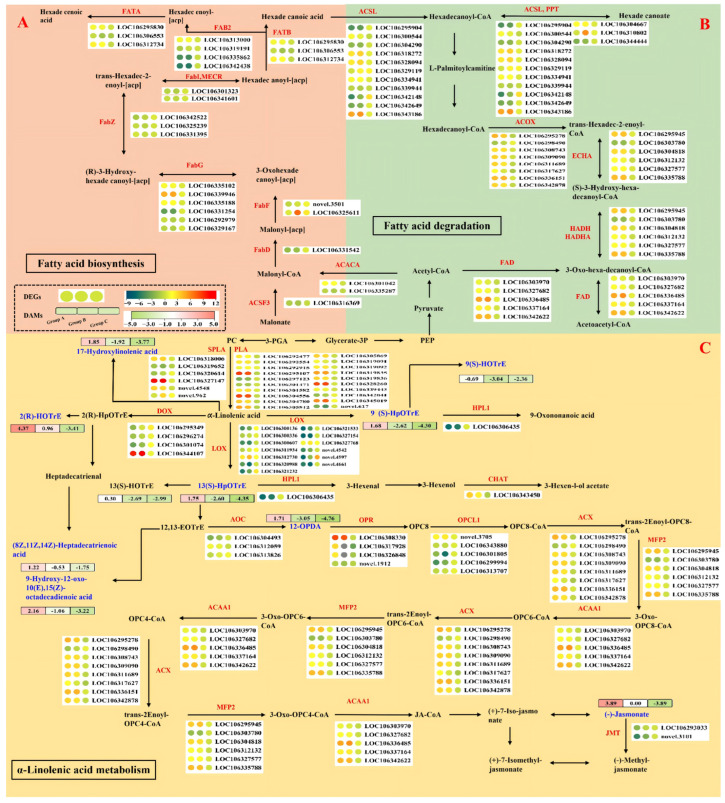
Differentially expressed genes (red) and DAMs (blue) related to “fatty acid biosynthesis” (ko00061, (**A**)), “fatty acid degradation” (ko00071, (**B**)), and “α-linolenic acid metabolism” (ko00592, (**C**)) in CK5 vs. CK0 (Group A), MT5 vs. CK0 (Group B), and MT5 vs. CK5 (Group C). FATA, fatty acyl-ACP thioesterase A (EC:3.1.2.14); FAB2, acyl-[acyl-carrier protein] desaturase (EC:1.14.19.2); FATB, fatty acyl-ACP thioesterase B (EC:3.1.2.14); ACSF3, malonyl-CoA/methylmalonyl-CoA synthetase (EC:6.2.1.76); ACSL, long-chain-fatty-acid-CoA ligase (EC:6.2.1.3); PPT, palmitoyl-protein thioesterase (EC:3.1.2.22); ACOX, acyl-CoA oxidase (EC:1.3.3.6); ECHA, enoyl-CoA hydratase (EC:4.2.1.17); HADH, 3-hydroxyacyl-CoA dehydrogenase (EC:1.1.1.35); HADHA, long-chain 3-hydroxyacyl-CoA dehydrogenase (EC:1.1.1.211); FAD, acetyl-CoA acyltransferase (EC:2.3.1.16); ACACA, acetyl-CoA carboxylase (EC:6.4.1.2); SPLA, secretory phospholipase A2 (EC:3.1.1.4); PLA, phospholipase A1 (EC:3.1.1.32); LOX, lipoxygenase (EC:1.13.11.12); CHAT, (Z)-3-hexen-1-ol acetyltransferase (EC:2.3.1.195); AOC, allene oxide cyclase (EC:5.3.99.6); OPR, 12-oxophytodienoic acid reductase (EC:1.3.1.42); ACAA, acetyl-CoA acyltransferase 1 (EC:2.3.1.16); JMT, jasmonate O-methyltransferase (EC:2.1.1.141); DOX, fatty acid alpha-dioxygenase (EC:1.13.11.92); FabZ,3-hydroxyacyl-[acyl-carrier-protein] dehydratase (EC:4.2.1.59); FabI, enoyl-[acyl-carrier protein] reductase I (EC:1.3.1.9); MECR, mitochondrial enoyl-[acyl-carrier protein] reductase/trans-2-enoyl-CoA reductase (EC:1.3.1.-); FabG,3-oxoacyl-[acyl-carrier protein] reductase (EC:1.1.1.100); FabF, 3-oxoacyl-[acyl-carrier-protein] synthase II (EC:2.3.1.179); FabD, [acyl-carrier-protein] S-malonyltransferase (EC:2.3.1.39); ACSF3, malonyl-CoA/methylmalonyl-CoA synthetase (EC:6.2.1.76); HPL1,hydroperoxide lyase (EC:4.1.2.-); OPCL1,OPC-8:0 CoA ligase 1 (EC:6.2.1.-); ACX, acyl-CoA oxidase (EC:1.3.3.6); MFP2,enoyl-CoA hydratase/3-hydroxyacyl-CoA dehydrogenase (EC:4.2.1.17); 2 (R)-HOTrE, 2(R)-hydroxy-9Z,12Z,15Z-octadecatrienoic acid; 2(R)-HpOTrE, 2(R)-hydroperoxy-9Z,12Z,15Z-octadecatrienoic acid; 9(S)-HpOTrE, 9(S)-hydroperoxy-9Z,12Z,15Z-octadecatrienoic acid; 13(S)-HOTrE, 13(S)-hydroxy-9Z,12Z,15Z-octadecatrienoic acid; 13(S)-HpOTrE, 13(S)-hydroperoxy-9Z,12Z,15Z-octadecatrienoic acid; PEP, Phosphoenolpyruvate; OPC8, OPC-8:0 CoA ligase 1; DAMs, differentially abundant metabolites; DEGs, differentially expressed genes.

This study identified 321 (19), 356 (10), and 126 (8) upregulated and 363 (5), 353 (20), and 58 (30) downregulated genes (metabolites) related to lipid metabolism in CK5 vs. CK0, MT5 vs. CK0, and MT5 vs. CK5, respectively (Table 1 and Figure 8). Further analysis indicated that “α-linolenic acid metabolism” and “fatty acid degradation” were the second and tenth enriched KEGG pathways for DEGs in CK5 vs. CK0, respectively. “Fatty acid elongation” was the eighth enriched KEGG pathway for DEGs in MT5 vs. CK5 (Tables S10–S12). “Sphingolipid metabolism” was the second, twelfth, and first enriched KEGG pathway for DAMs in CK5 vs. CK0, MT5 vs. CK0, and MT5 vs. CK5, respectively (Tables S18–S20). “Lipid catabolic process” and “lipid oxidation” were the seventh and eleventh enriched GO terms in BP for CK5 vs. CK0 (Table S7).

This study identified 28 (zero), 28 (zero), and 6 (zero) upregulated and 53 (zero), 56 (zero), and 10 (zero) downregulated genes (metabolites) related to chlorophyll metabolism in CK5 vs. CK0, MT5 vs. CK0, and MT5 vs. CK5, respectively (Table 1).

Phospholipids are the backbone of biological membranes and play a role in signal transduction. Phospholipases cleave diverse bonds in PLs, thereby producing cell signals, such as FFAs, phosphatidic acid (PA), LPLs, and diacylglycerol (DAG) [38,39]. Lipoxygenases catalyze the oxidation of polyunsaturated FAs to yield peroxide products, MDA, and free radicals, thereby causing membrane damage and accelerating membrane lipid degradation [14]. Table 2 lists the differentially expressed phospholipase and LOX genes identified in CK5 vs. CK0, MT5 vs. CK0, and/or MT5 vs. CK5. Additionally, Table 3 and Table 4 list some of the DEGs involved in “FA β-oxidation” (peroxisomal membrane protein, and peroxisomal citrate synthase genes) and chlorophyll degradation (magnesium-dechelatase (SGR), pheophorbide a oxygenase (PAO), red chlorophyll catabolite reductase (RCCR), and pheophorbidase (PPD)), respectively.

## 3. Discussion

### 3.1. Static Magnetic Field Delays Broccoli Senescence by Reducing Chlorophyll Degradation

The research showed that 5 mT SMF inhibited the upregulation of floral organ senescence-related genes in broccoli florets during storage (Table 1) and that 5 mT SMF delayed the yellowing of broccoli florets during storage (Figure 1), indicating that 5 mT SMF delayed the senescence of broccoli florets during storage. Postharvest yellowing of broccoli florets is the most apparent feature of quality deterioration, directly affecting their commercial quality. The results revealed that 5 mT SMF significantly delayed the increases in L*, a* and b* values and yellowing in postharvest broccoli compared with the control (Figure 1 and Figure 2), indicating that 5 mT SMF effectively delayed the postharvest yellowing of broccoli florets. This agreed with the report that MF delayed yellowing of green chili during storage [40].

Chloroplasts are one of the earliest catabolic sites in leaf senescence [41]. The breakdown of chlorophyll during senescence is a crucial stage in plant development, converting the green pigment into colorless compounds. The first step in the degradation of chlorophyll is the removal of central magnesium (Mg) by Mg-dechelatase (SGR). It is a rate-limiting step in the chlorophyll breakdown pathway [42]. Additionally, pheophorbide a oxygenase (PAO; EC:1.14.15.17), red chlorophyll catabolite reductase (RCCR; EC:1.3.7.12), and pheophorbidase (PPD; EC:3.1.1.82) are involved in the degradation of chlorophylls [43,44]. Melatonin suppressed the activity of RCCR to delay the yellowing of broccoli [45]. The research detected 12 upregulated (three SGR + one PAO + one RCCR + seven PPD) genes and three downregulated PPD genes in CK5 vs. CK0; nine upregulated (three SGR + two PAO + four PPD) genes and five downregulated (one SGR + one PAO + three PPD) genes in MT5 vs. CK0; and one upregulated and four downregulated PPD genes in MT5 vs. CK0 (Table 3). Under stress conditions, chloroplasts serve as the primary site for ROS production and are also the targets of ROS-induced damage [46]. Oxidative damage can lead to a decline in pigment concentration [47]. Regression analysis showed that total chlorophyll content was significantly negatively related to MDA content (Figure 4). These results suggested that 5 mM SMF prevented the upregulation of chlorophyll degradation-related genes and the increase in oxidative damage (MDA content) in broccoli florets during storage, thereby reducing the breakdown of chlorophylls and delaying postharvest yellowing and senescence (Figure 1, Figure 2 and Figure 3).

### 3.2. Static Magnetic Field Delayed Postharvest Yellowing and Senescence of Broccoli Florets by Enhancing the Ability of Postharvest Broccoli Florets to Maintain Plasma Membrane Integrity

Lipids are one of the principal components of cell membranes, play irreplaceable roles in maintaining membrane structure, regulating fluidity, mediating stress responses, and fruit wax formation [13]. The destruction of membrane integrity is highly related to fruit and vegetable senescence and deterioration during storage, which is caused by the degradation of membrane components and lipid peroxidation [14,46]. Electrolyte leakage is a key indicator for evaluating the severity of membrane damage and is related to the quality and shelf life of vegetables and fruit [46,47]. Increased lipid peroxidation and electrolyte leakage have been observed in postharvest vegetables and fruit during storage [10,15,48,49]. The research indicated that electrolyte leakage was significantly positively related to MDA content (Figure 4), indicating that 5 mT SMF alleviated the increase in lipid peroxidation (MDA content) in broccoli florets during storage, thereby lowering electrolyte leakage (Figure 3). This was supported by the previous findings that MF delayed postharvest increases in MDA content and electrolyte leakage of strawberry fruit [22], cherry tomato fruit [25], and cucumber [23].

Phospholipids are the essential components and the structural basis of biological membranes [50] and play a key role in controlling membrane fluidity and permeability [51]. Sterols are the membrane components and regulate the fluidity and the permeability of PL bilayers [52]. A high ratio of PLs to sterols is associated with elevated membrane fluidity [53]. It was observed that during rose petal senescence, the concentrations of PLs reduced without any significant alteration in the concentrations of free sterols, and the fluidity of the rose petal membrane decreased with age due to a reduction in PL concentration and an increase in the ratio of free sterols to PLs [54]. The research detected one downregulated PC in CK5 vs. CK0; one upregulated PC in MT5 vs. CK5; and two, one, and two upregulated and four, four, and two downregulated steroids in CK5 vs. CK0, MT5 vs. CK0, and MT5 vs. CK5, respectively (Figure 6E–J). Further analysis suggested that the decrease in PLs (PC) in CK5 vs. CK0 was caused by both increased catabolism and decreased biosynthesis (Table 1), and the increase in PC in MT5 vs. CK5 was caused by increased biosynthesis, as indicated by more upregulated than downregulated genes involved in the “PL biosynthesis process” (Table 1) rather than by decreased degradation, as indicated by more upregulated than downregulated genes involved in the “PL catabolic process” (Table 1) and five upregulated and two downregulated phospholipase genes (Table 2). Additionally, the decrease in steroids in CK5 vs. CK0 and MT5 vs. CK0 was caused by decreased biosynthesis (Table 1). This agreed with the report that during rose petal senescence, the reduction in membrane PLs was brought about by both elevated degradation and decreased biosynthesis [54]. These results suggested that 5 mT SMF prevented the decrease in PL abundances and increased the ratio of PLs to sterols in broccoli florets during storage, thereby maintaining membrane fluidity and stability.

Phospholipids can be hydrolyzed by multiplex phospholipases. Phospholipase As (PLAs) cleave sn-1 and sn-2 positions of glycerophospholipids to produce FFAs and LPLs [38,39]. Zhang et al. [55] observed that the TtPLA1−1 (from salt-tolerant Tritipyrum “Y1805”) overexpression wheat lines had higher concentrations of LPLs and FAAs than the wild-type plants. The research identified 16 upregulated and four downregulated PLAs in CK5 vs. CK0; 15 upregulated and three downregulated PLAs in MT5 vs. CK0; and one upregulated PLA1 in MT5 vs. CK5 (Table 2). The upregulation of PLAs might contribute to the increase in the abundances of LPEs in CK5 vs. CK0 as well as LPCs and LPEs in MT5 vs. CK0 and MT5 vs. CK5, whereas the decrease in the abundances of LPCs in CK5 vs. CK0 (six increased and 11 decreased LPCs) might be brought about by the decrease in the abundance of PC (Figure 6H–J). Lysophospholipids are minor membrane components of many tissues and have been exogenously applied to delay the senescence of leaves, flowers, and fruit in various plants, including snapdragon flowers [56], Philodendron cordatum (Vell.) Kunth leaves [57], tomato leaves and fruit [58], potato (*Solanum tuberosum* L.,) leaves [59], and banana fruit (excised peel) [60], through lowering water loss and electrolyte leakage; inhibiting ethylene production, lipid degradation, and phospholipase D (PLD) activity (an enhancer of senescence progression) [61,62]; and delaying chlorophyll degradation. These results suggested that 5 mT SMF increased the abundances of LPLs in broccoli florets during storage, thereby delaying their postharvest senescence.

Sphingolipids are the components of cell membranes [63] and play a role in keeping membrane structural integrity and function [64]. Sphingolipids with a simpler structure, such as ceramides and long-chain bases, can serve as signaling molecules and function in cellular pathways [64] and serve as a powerful supporter to help the membrane deal with abiotic stresses [63]. Zhao et al. [65] observed that exogenous application of ceramide reduced water loss and MDA content and delayed the rot of strawberry fruit. The research obtained six and 11 increased sphingolipids in CK5 vs. CK0 and MT5 vs. CK0, respectively, as well as six upregulated and six downregulated sphingolipids in MT5 vs. CK5 (Figure 6H–J). These findings suggested that 5 mT SMF led to an increase in sphingolipids in broccoli florets during storage, thereby reducing oxidative stress and keeping membrane integrity.

Taken together, 5 mT SMF increased the abundances of LPLs and sphingolipids and prevented the decrease in PC abundance in broccoli florets during storage, thereby enhancing the ability of postharvest broccoli florets to produce energy, reducing oxidative stress, and maintaining plasma membrane integrity.

### 3.3. Static Magnetic Field Delayed Postharvest Yellowing and Senescence of Broccoli Florets Through Regulating Lipid Catabolism and Alleviating Senescence-Associated Energy Imbalance

The research indicated that 5 mT SMF mitigated the changes in lipid oxidation in broccoli florets during storage, as indicated by more DEGs related to “FA β-oxidation” in CK5 vs. CK0 than in MT5 vs. CK0 (Table 1). The research detected five upregulated (one peroxisomal membrane protein 2 + four peroxin) genes and six downregulated peroxin genes in CK5 vs. CK0; eight upregulated (one peroxisomal membrane protein 2 + seven peroxin) genes and three downregulated peroxin genes in MT5 vs. CK0; and three upregulated (one peroxisomal membrane protein 2 + two peroxin) genes and one downregulated peroxin gene in MT5 vs. CK5 (Table 4). Peroxisome function and formation are coordinated by peroxins and peroxisomal membrane proteins that guide peroxisome biogenesis [66]. Peroxisome biogenesis proteins are essential for peroxisomal membrane integrity, whose upregulation ensures de novo peroxisome biogenesis and physical integrity of the peroxisomal membrane, thus preventing the release of oxidative enzymes into the cytosol but detoxifying them within the organelle [67]. These results suggested that 5 mT SMF upregulated the expression of peroxisome biogenesis protein genes in broccoli florets during storage, thereby preventing peroxisome biogenesis disorders (PBDs) caused by defects in peroxisome biogenesis and maintaining membrane integrity [68].

The breakdown of FAs by peroxisomal β-oxidation helps plants to maintain metabolic and energy homeostasis under carbon-starved conditions through providing energy and carbon skeletons [69]. Notably, the β-oxidation activation is associated with the accumulation of adenosine 5′-monophosphate (AMP), which reflects cellular ATP demand under carbon starvation [70]. The metabolomic profiling revealed the increased abundance of adenosine 5′-monophosphate (AMP, pmb0981) in MT5 vs. CK0 and MT5 vs. CK5 (Tables S14 and S15), which serves as an upstream signal to activate catabolic processes, including fatty acid β-oxidation, to restore ATP homeostasis in the MT group. The study obtained three upregulated (two citrate synthase 2, peroxisomal (CISY2) + one CISY3) genes and one downregulated CISY2 gene in CK5 vs. CK0; four upregulated (three citrate CISY2 + one CISY3) genes and one downregulated CISY2 in MT5 vs. CK0; and one upregulated CISY2 in MT5 vs. CK5 (Table 4). The complete respiration of FAs requires oxidation of the acetyl-coenzyme A (CoA) biosynthesized by peroxisomal β-oxidation. Peroxisomal citrate synthases catalyze the conversion of acetyl-CoA to citrate. The yielded citrate can be exported from the peroxisome for subsequent respiration in the mitochondrion to maintain ATP biosynthesis [71]. A study indicated that both ATP concentration and energy charge were reduced during the postharvest senescence of litchi fruit, and there was a significant negative relationship between ATP concentration and fruit browning index [72]. It was observed that exogenous application of ATP effectively delayed postharvest senescence and maintained the fruit quality of longan fruit by maintaining higher ATP and ADP concentrations, higher energy charge, lower respiration, lower weight loss rate, and lower electrolyte leakage [73]. Tert-Butylhydroquinone-mediated alleviation of longan fruit deterioration during storage an involved enhanced ability to maintain higher ATP and ADP concentrations and a higher energy charge [48]. Liu et al. [22] reported that MF-mediated delaying of strawberry fruit senescence during storage involved enhanced ATP concentration and energy charge. Wei et al. [40] indicated that MF delayed the quality alterations by enhancing ATP concentration and energy charge in green chili during storage. The more upregulated CISYs in MT5 vs. CK0 than in CK5 vs. CK0 and the upregulation of CISY2 in MT5 vs. CK5 suggested that 5 mT SMF upregulated the expression of peroxisomal citrate synthase genes in broccoli florets during storage, thereby (a) increasing peroxisome to mitochondrion citrate transport; (b) maintaining higher ATP biosynthesis, lower weight loss, and lower electrolyte leakage; and (c) delaying postharvest senescence and yellowing of broccoli florets.

The research detected 66 increased (22 saturated + 44 unsaturated) and seven decreased (one saturated + six unsaturated) FFAs in CK5 vs. CK0; 29 increased (16 saturated + 13 unsaturated) and 52 decreased (eight saturated + 44 unsaturated) FFAs in MT5 vs. CK0; and eight increased (three saturated + five unsaturated) and 89 decreased (22 saturated + 67 unsaturated) FFAs in MT5 vs. CK5 (Figure 6E–J and Table S17). The increase in the abundances of saturated FFAs in CK5 vs. CK0 was mainly caused by the release of saturated FAs from membrane lipids brought about by upregulated PLAs (Table 2) rather than by increased biosynthesis of FAs, as indicated by more downregulated than upregulated genes related to “FA biosynthesis” and “FA biosynthesis process”, and decreased degradation of catabolism and β-oxidation, as indicated by more upregulated than downregulated genes involved in “FA degradation”, “FA catabolic process”, “lipid oxidation”, and “FA β-oxidation” (Table 1). However, the increase in the abundances of unsaturated FFAs in CK5 vs. CK0 might be caused by both increased release of unsaturated FAs from membrane lipids brought about by upregulated PLAs (Table 2) and increased biosynthesis, as indicated by more upregulated than downregulated genes related to “biosynthesis of unsaturated FAs” in CK5 vs. CK0 (Table 1). Lipoxygenases catalyze the oxygenation of polyunsaturated FAs [74]. The increase in the levels of FFAs (Figure 6E,H) and MDA (Figure 3A) and the downregulation of LOXs (Table 2) in CK5 vs. CK0 agreed with the reports that with the increasing severity of cold stress in non-acclimated wheat plants, the activity of LOX reduced along with an increment in MDA level to help increase or maintain the levels of unsaturated FAs [74]; the increase in MDA content was not necessarily directly related to the increase in LOX activity [75]; and lipid peroxidation was partially brought about by the increased availability of unsaturated FFA substrates for LOX [75]. The increases in FFAs (Figure 6H), MDA content, and electrolyte leakage (Figure 3A,B) suggested that the membrane damage in CK5 vs. CK0 might be induced by ATP limitation [69].

The decrease in FFAs in MT5 vs. CK0 and MT5 vs. CK5 (Figure 6I,J) suggested that the β-oxidation and degradation of FAs exceeded the biosynthesis of FAs from membrane lipids [76]. Further analysis indicated that the increase in saturated FFAs in MT5 vs. CK0 was mainly caused by increased release of saturated FAs from membrane lipids brought about by upregulated expression of PLAs (Table 2), and the decrease in unsaturated FFAs in MT5 vs. CK0 was caused by increased catabolism and oxidation, as indicated by more upregulated than downregulated genes related to the “FA catabolic process” and “FA oxidation” (Table 1). The decrease in saturated and unsaturated FAAs in MT5 vs. CK5 was brought about by increased degradation, as indicated by more upregulated than downregulated genes related to “FA catabolic process”, “FA oxidation”, and “FA β-oxidation” (Table 1) and six upregulated LOXs (Table 2, Figure 8). The decreases in electrolyte leakage and MDA content (Figure 3A,B) and the upregulation of LOXs (Table 2) in MT5 vs. CK5 (Figure 3A,B) agreed with the reports that lipid peroxidation was partially due to the elevated availability of FFAs for LOX [76], and an increase in LOX activity did not necessarily mean an increase in MDA level [75].

During the senescence of organs (leaves), most FAs are either oxidized or converted to α-ketoglutarate via the glyoxylate cycle to supply the energy required for senescence [76]. The decrease in FFAs in MT5 vs. CK0 and MT5 vs. CK5 could be partially explained by the increased FA β-oxidation (Table 1) and/or conversion of FFAs to α-ketoglutarate because the abundance of α-ketoglutarate increased in MT5 vs. CK5 (Table S15). Acting as an intermediate metabolite of tricarboxylic acid cycle, α-ketoglutarate is required for energy generation. It can detoxify ROS via its reaction with H_2_O_2_ during the tricarboxylic acid cycle to produce H_2_O, succinate, and CO_2_ [77]. α-Ketoglutarate-mediated alleviation of arsenate toxicity in Solanum melongena L. involved reduced oxidative damage and an enhanced ability to keep membrane stability [78]. The decrease in FFAs in MT5 vs. CK0 and MT5 vs. CK5 suggested that 5 mT SMF enhanced the ability of postharvest broccoli florets to alleviate senescence-associated energy imbalances.

In conclusion, SMF prevented peroxisome biogenesis disorders (PBDs) and maintained peroxisomal membrane integrity by upregulating the expression of peroxisome biogenesis protein genes in broccoli florets during storage. Enhanced FA β-oxidation and TCA cycle-related metabolite turnover might alleviate senescence-associated energy imbalance, oxidative damage, weight loss and electrolyte leakage, thereby delaying yellowing and senescence in broccoli florets during postharvest storage.

### 3.4. Static Magnetic Field Enhanced the Ability of Postharvest Broccoli Florets to Prevent Water Loss

Epidermal lipids (cutin, suberin, and wax) function as a protective barrier between the fruit and the external environment, thereby preventing water loss and pathogen infection, protecting against biotic and abiotic stresses and maintaining postharvest performance during storage [79,80,81]. The research obtained 15 (one), 23 (zero), and 14 (zero) upregulated and 31 (zero), 25 (two), and three (three) downregulated genes (metabolites) related to “cutin, suberine and wax biosynthesis” in CK5 vs. CK0, MT5 vs. CK0, and MT5 vs. CK5, respectively. The research identified more downregulated than upregulated genes related to the “cutin biosynthetic process” and the “wax biosynthetic process” and more upregulated than down-regulated genes related to the “suberin biosynthetic process” in CK5 vs. CK0 and MT5 vs. CK0, but there were more upregulated than downregulated genes related to the three GO terms in MT5 vs. CK5 (Table 1). These results suggested that MT5 increased the biosynthesis of cutin, suberin, and wax in broccoli florets during storage relative to CK5, thereby preventing water (weight) loss (Figure 3C).

Phospholipase Ds hydrolyze the terminal phosphodiester bonds of PLs to generate the soluble head group and phosphatidic acid and play a role in dehydration and drought tolerance in plants [39,82]. Using PLDa-overexpressing tobacco and PLDa-depleted Arabidopsis as materials, Sang et al. [83] observed that the PLDa-depleted Arabidopsis leaves had a higher transpiration water loss, brought about by impaired stomatal closure and reduced sensitivity to ABA, and a lower drought tolerance than the WT plants. However, PLDa-overexpressing tobacco leaves had a lower transpiration water loss brought about by enhanced stomatal closure and increased sensitivity to ABA. The study obtained zero, one, and one downregulated and five, nine, and four upregulated PLD genes in CK5 vs. CK0, MT5 vs. CK0, and MT5 vs. CK5, respectively (Table 2). These results suggested that 5 mT SMF promoted the upregulation of PLDs in broccoli florets during storage, thereby preventing water loss.

## 4. Materials and Methods

### 4.1. Treatment and Sampling

Broccoli (*Brassica oleracea* L. var. italica) heads were harvested at commercial maturity from local producers in Longhai (24°24′ N, 117°57′ E), Zhangzhou, Fujian Province, China, when florets presented dark green, compact inflorescence, and flower buds were closed, approximately 12–15 cm in diameter and 0.35–0.4 kg in weight each. The harvested broccoli heads were immediately transported to the laboratory in Zhangzhou Institute of Technology within 1.5 h. Uniformly colored broccoli heads without mechanical damage, disease, or insect infestation were selected as experimental materials.

Data from preliminary experiments showed that the effect of SMF on delaying the yellowing of broccoli was enhanced by increasing SMF strength in the range of 0–5 mT and then remained stable between 5 and 10 mT. Therefore, the subsequent experiments were carried out under an SMF intensity of 5 mT, balancing personnel safety and energy efficiency [84,85]. After pre-cooling at 4 °C for 3 h, 120 hand-harvested broccoli heads were randomly assigned to two groups (MT group and control (CK) group). All treated broccoli heads were packaged in 0.03 mm polyethylene bags with the top folded over to decrease water loss. Both the control and SMF-treated (MT) groups were stored in MF refrigerators of the same model (INDUC Science Co., Ltd., Wuxi, China), with the MF intensity set at 0 mT and 5 mT, respectively. Preservation was performed at 20 °C with 90% relative humidity (RH) and monitored every day during storage. Florets were excised from 10 broccoli heads prior to treatment (day 0) and daily during storage for 5 days; immediately frozen in liquid nitrogen; and preserved at −80 °C until extraction of chlorophylls, MDA, RNA, and metabolites.

### 4.2. Determination of Phenotypic Color and Total Chlorophyll Content

A colorimeter (ColorQuest XE, HunterLab, Reston, VA, USA) was used to evaluate the color characteristics of broccoli. For each replicate, ten broccoli heads were randomly chosen, and color measurements were taken at five different positions on each head, with each location measured three times. The color parameters were recorded as L* (L* = 0 corresponded to black; L* = 100 corresponded to white), a* (negative values represented green and positive values denoted red), and b* (negative indicated blue and positive indicated yellow).

The chlorophyll content was quantified following the protocol of Sun et al. [85]. Briefly, about 2 g of broccoli floret flesh was homogenized in 10 mL of acetone and centrifuged at 4 °C for 15 min at 10,000 g. The absorbance of the supernatant was measured at 647 nm and 665 nm with a SuperMax 3000FA multi-mode microplate reader (Shanghai Flash Spectrum Biological Technology Co., Ltd., Shanghai, China). Chlorophyll content (g kg^−1^) was calculated as follows:

$$\mathrm{Total}\;\mathrm{chlorophyll}\;\mathrm{content}\;(g\;{\mathrm{kg}}^{-1})=(17.90\times A_{647}+8.08\times A_{665})\times V/m$$

where V was the total volume of the extract solution (mL), and m represented the sample mass (g).

### 4.3. Determination of MDA Content

The malondialdehyde (MDA) content was determined based on the method described by Fang et al. [10]. Briefly, 1 g of fresh broccoli florets was homogenized in 5 mL of 10% (*w*/*v*) trichloroacetic acid (TCA) solution. The homogenate was centrifuged at 12,000× *g* for 30 min at 4 °C to collect the supernatant. Subsequently, 2 mL of the collected supernatant was mixed with 2 mL of 0.67% (*w*/*v*) thiobarbituric acid (TBA), and the mixture was kept in boiling water for 15 min. After cooling to room temperature, the mixture was centrifuged at 10,000× *g* for 15 min. The absorbance of the supernatant was measured with a spectrophotometer (Model DDSJ-318T, Leici Inc., Shanghai, China) at 450, 532, and 600 nm. Malondialdehyde content was calculated as follows:

$$\mathrm{MDA}\;(\mathrm{mmol}\;{\mathrm{kg}}^{-1})=[6.45\times (A_{532}-A_{600})-0.56\times A_{450}]\times \mathrm{Vt}/(\mathrm{Vs}\times m)$$

where, Vt, Vs, and m denote the total volume of the extract (mL), the volume of extract used in the assay (mL), and the flesh sample mass (g), respectively.

### 4.4. Measurement of Electrolyte Leakage

Electrolyte leakage assessment was performed as outlined by Sun et al. [86]. About 0.5 g was transferred to a 100 mL Erlenmeyer flask, and 25 mL distilled water was subsequently added. After being equilibrated at 25 °C for 30 min, the initial electrical conductivity (P1) was measured with a conductivity meter (Model DDSJ-318T, Leici Inc., Shanghai, China). The mixture was boiled for 20 min, then the final electrical conductivity (P2) was determined after cooling to room temperature. The relative electrolyte leakage was calculated as follows:

$$\mathrm{Electrolyte}\;\mathrm{leakage}\;(\%)\;=\frac{P1}{P2}\times 100\%$$

### 4.5. Measurement of Weight Loss

For each experimental group, five broccoli heads were selected, and their initial mass (W0) was recorded prior to storage. During the entire storage period, the mass (Wn) of each labeled broccoli head was weighed daily to calculate the weight loss (%). The weight loss rate was calculated as follows:

$$\mathrm{Weight}\;\mathrm{loss}\;(\%)=\frac{W0-\mathrm{Wn}}{W0}\times 100\%$$

### 4.6. Transcriptome and Widely Targeted Metabolome in Broccoli Florets

Broccoli florets collected at 0 d and 5 d after SMF treatments were subjected to transcriptomic and metabolomic profiling. A total of nine frozen samples, namely three biological replicates for CK0 (broccoli florets collected prior to treatment, day 0), CK5 (broccoli florets collected on the fifth day with 0 mT magnetic field intensity), and MT5 (broccoli florets collected on the 5/5 day with 5 mT magnetic field intensity).

RNA extraction, reverse transcription, sequencing library construction, and RNA sequencing were conducted by Wuhan MetWare Biotechnology Co. Ltd., Wuhan, China (www.metware.cn, accessed on 8 October 2024). The obtained raw reads were filtered using fastp with the following parameters: (1) adapter-containing reads were excluded; (2) paired reads were excluded from subsequent analyses if the proportion of N bases in either read surpassed 10%; and (3) paired reads were excluded when low-quality bases (Q ≤ 20) in either read accounted for more than 50% of the total base number. The yielded clean reads were mapped to the reference genome (https://www.ncbi.nlm.nih.gov/datasets/genome/GCF_000695525.1/, accessed on 24 October 2024) of broccoli using HISAT 2v2.2.1..

Differential gene expression was analyzed with the DESeq2 package, and *p*-values were adjusted using Benjamini and Hochberg’s method. Adjusted *p*-values (padj) ≤ 0.05 and|log2fold change (FC)|≥ 1 were set as the threshold for defining differentially expressed genes (DEGs).

Enrichment analysis was conducted based on the hypergeometric test, with KEGG pathways and GO terms analyzed separately. For KEGG, pathway-level enrichment was assessed via hypergeometric distribution testing, while GO terms were evaluated using term-specific analysis.

A widely-targeted metabolome was carried out by Wuhan MetWare Biotechnology Co., Ltd. on the UPLC-ESI-MS/MS system (UPLC, ExionLC™ AD, Marsiling Industrial Estate, Woodlands, Singapore; MS, Applied Biosystems 4500 Q TRAP, Marsiling Industrial Estate, Woodlands, Singapore). Briefly, 50 mg of floret powder was mixed with 1.2 mL of a 70% methanolic aqueous solution containing the internal standard. The mixture was vortexed for 30 s every 30 min (six times total) and then centrifuged at 16,000× *g* for 3 min. The supernatant was filtered through a microporous membrane (0.22 μm pore size) and then loaded on an UPLC–ESI–MS/MS system (UPLC, ExionLC™ AD, https://sciex.com.cn/) and a Tandem mass spectrometry system (https://sciex.com.cn/).

The mobile phase was composed of 0.1% formic acid in water (A) and 0.1% formic acid in acetonitrile (B). The gradient elution with a flow rate of 0.35 mL min-1 was as follows: the proportion of phase B was maintained at 5% at 0.00 min. Subsequently, the proportion of phase B was linearly gradient to 95% within 9.00 min and held at this level for 1 min. From 10.00 min to 11.10 min, the proportion of phase B decreased back to 5%, which was then maintained for re-equilibration until 14.00 min. The column oven was set to 40 °C with an injection volume of 2 μL. The effluent was directly coupled to an ESI-Q TRAP-MS/MS.

For two-group comparisons, differentially abundant metabolites (DAMs) were screened based on|Log2FC|≥1.0 and VIP (variable importance in projection) >1 extracted from orthogonal partial least squares-discriminant analysis (OPLS-DA). Functional annotation of DAMs was performed with the MetWare metabolite database, KEGG Compound database (http://www.kegg.jp/kegg/compound/, accessed on 1 November 2025), and KEGG Pathway database (http://www.kegg.jp/kegg/pathway.html, accessed on 1 November 2025).

### 4.7. RT-qPCR Verification

The transcriptome data were validated via RT-qPCR analysis. Ten genes involved in “α-linolenic acid metabolism” (LOC106312730), “cutin, suberine and wax biosynthesis” (LOC106332210, LOC106299122, LOC106327484), “glycerophospholipid metabolism” (LOC106295624), “sphingolipid metabolism” (LOC106328575 and LOC106317133), “glycerolipid metabolism” (LOC106301685), “fatty acid elongation” (LOC106295733), and “fatty acid biosynthesis” (LOC106342148) were selected. There were three biological replicates × three technical replicates for each treatment. The actin served as an internal control. Table S1 listed the primer sequences designed with Primer PREMIER 5.0 (Premier Biosoft International, Palo Alto, CA, USA). The relative gene expression levels were quantified via the 2^–ΔΔCT^ method.

### 4.8. Integration of Metabolome and Transcriptome

Integrated analysis involved correlation of metabolite and gene levels, KEGG pathway enrichment, and network construction to link metabolic and transcriptional changes. The data for conjoint analysis were filtered according to Pearson’s correlation coefficients (PCCs) > 0.80 and the corresponding *p*-values < 0.05.

### 4.9. Statistical Analysis

Three biological replicates were included for each experiment, with results shown as mean ± SD. Statistical analyses were conducted using DPS by the one-way ANOVA and Duncan’s multiple range test to identify differences, where *p* < 0.05 was considered significant. Calculation of PCCs, principal component analysis (PCA), and hierarchical cluster analysis (HCA) were performed by R package (version 3.50).

## 5. Conclusions

The current results demonstrated that 5 mT SMF delayed postharvest senescence of broccoli florets through the coordination of multiple factors: (1) downregulating the expression of floral organ senescence-related genes; (2) slowing down the breakdown of chlorophylls through preventing the upregulation of chlorophyll degradation-related genes and the increase in oxidative stress; (3) alleviating senescence-associated energy imbalance through enhancing fatty acid β-oxidation and peroxisomal metabolic flux to reduce water loss and oxidative stress and maintain membrane integrity; (4) increasing the abundances of LPLs and sphingolipids and preventing the decrease in PC abundance to lower water loss and oxidative stress, inhibit ethylene production, delay chlorophyll degradation, and keep membrane integrity; (5) reducing water loss via increasing cutin, suberin, and wax biosynthesis and stomatal closure brought about by upregulated expression of PLDs; and (6) preventing the increase in MDA content, electrolyte leakage, and weight loss rate. As a result, these findings offer novel insights into the underlying methods by which the SMF delayed the postharvest senescence of broccoli florets and a scientific basis for the application of SMF in postharvest storage of broccoli florets. The application of SMF presents a promising strategy for maintaining the postharvest quality of broccoli florets by delaying senescence and prolonging shelf life.

## Figures and Tables

**Table 1:** Differentially expressed genes and DAMs related to senescence, lipid metabolism and chlorophyll metabolism in CK5 vs. CK0, MT5 vs. CK0, and/or MT5 vs. CK5.

Description	CK5 vs. CK0 DEGs (DAMs)		MT5 vs. CK0 DEGs (DAMs)		MT5 vs. CK5 DEGs (DAMs)	
	Up	Down	Up	Down	Up	Down
Senescence						
Floral organ senescence (GO:0080187)	7	2	6	4	1	3
Plant organ senescence (GO:0090693)	123	59	126	55	28	22
Leaf senescence (GO:0010150)	118	53	121	52	24	21
Total	123	59	126	55	28	22
Lipid metabolism						
Fatty acid degradation (ko00071)	39	15	39	12	3	2
Fatty acid elongation (ko00062)	5	14	8	13	8	3
Fatty acid biosynthesis (ko00061)	12	16	13	24	3	2
Fatty acid biosynthetic process (GO:0006633)	45	69	49	75	31	12
Fatty acid catabolic process (GO:0009062)	44	8	41	3	4	1
Fatty acid oxidation (GO:0019395)	43	12	40	7	4	1
Fatty acid β-oxidation (GO:0006635)	41	7	38	3	2	1
Glycerolipid metabolism (ko00561)	42	32 (2)	45 (1)	30	10 (2)	2
Glycerophospholipid metabolism (ko00564)	57	41 (2)	63 (2)	43	20 (3)	4
Sphingolipid metabolism (ko00600)	15 (4)	16	16 (4)	16	6 (1)	1 (4)
Sphingolipid biosynthetic process (GO:0030148)	10	8	12	6	2	0
Ether lipid metabolism (ko00565)	16	6(1)	20	6	5(1)	1
Steroid biosynthesis (ko00100)	12	23	14	22	2	5
Steroid biosynthetic process (GO:0006694)	14	43	18	43	8	9
Linoleic acid metabolism (ko00591)	6 (6)	13	8 (3)	10 (11)	8 (1)	1 (13)
α-Linolenic acid metabolism (ko00592)	45 (8)	29	40	22 (7)	11	3 (10)
Biosynthesis of unsaturated fatty acids (ko01040)	13	7	11	5	0	1
Arachidonic acid metabolism (ko00590)	5	4	7	5	1	0
Cutin, suberin and wax biosynthesis (ko00073)	15 (1)	31	23	25 (2)	14	3 (3)
Cutin biosynthetic process (GO:0010143)	17	23	15	24	5	4
Suberin biosynthetic process (GO:0010345)	18	14	19	8	7	5
Wax biosynthetic process (GO:0010025)	13	32	14	30	12	3
Membrane lipid biosynthetic process (GO:0046467)	18	25	25	26	8	1
Phospholipid biosynthetic process (GO:0008654)	48	56	55	63	18	5
Phospholipid catabolic process (GO:0009395)	11	4	15	5	6	1
Lipid catabolic process (GO:0016042)	135	75	139	62	37	17
Lipid oxidation (GO:0034440)	47	28	45	21	12	2
Glyoxylate cycle (GO:0006097)	7	3	7	3	0	0
Total	321 (19)	363 (5)	356 (10)	353 (20)	126 (8)	58 (30)
Chlorophyll metabolism						
Chlorophyll biosynthetic process (GO:0015995)	13	47	13	49	2	9
Chlorophyll catabolic process (GO:0015996)	15	6	15	7	4	1
Total	27	53	27	56	6	10

**Table 2:** Differentially expressed phospholipase and lipoxygenase (LOX) genes identified in CK5 vs. CK0, MT5 vs. CK0, and/or MT5 vs. CK5.

Accession No.	KEGG	log_2_ (FC)		
		Group A	Group B	Group C
Phospholipases				
LOC106292554	K22389 phospholipase A1 [EC:3.1.1.32]|(RefSeq) phospholipase A(1) LCAT3 (A)	2.493	2.537	
LOC106301471	K22389 phospholipase A1 [EC:3.1.1.32]|(RefSeq) phospholipase A(1) LCAT3 (A)	−1.431	1.474	
LOC106305812	K22389 phospholipase A1 [EC:3.1.1.32]|(RefSeq) lecithin-cholesterol acyltransferase-like 4 (A)	5.167	4.180	
LOC106305869	K22389 phospholipase A1 [EC:3.1.1.32]|(RefSeq) lecithin-cholesterol acyltransferase-like 4 (A)	3.320	3.077	
novel.617	K22389 phospholipase A1 [EC:3.1.1.32]|(RefSeq) lecithin-cholesterol acyltransferase-like 4 (A)	−3.008	−4.761	
LOC106292477	K16818 phospholipase A1 [EC:3.1.1.32]|(RefSeq) phospholipase A(1) DAD1, chloroplastic (A)	2.474	1.993	
LOC106292915	K16818 phospholipase A1 [EC:3.1.1.32]|(RefSeq) phospholipase A(1) DAD1, chloroplastic (A)	1.035	1.929	
LOC106295107	K16818 phospholipase A1 [EC:3.1.1.32]|(RefSeq) phospholipase A(1) DAD1, chloroplastic (A)	8.918	8.033	
LOC106297123	K16818 phospholipase A1 [EC:3.1.1.32]|(RefSeq) phospholipase A(1) DAD1, chloroplastic (A)	−3.127		
LOC106301582	K16818 phospholipase A1 [EC:3.1.1.32]|(RefSeq) phospholipase A(1) DAD1, chloroplastic (A)	2.386	2.847	
LOC106304556	K16818 phospholipase A1 [EC:3.1.1.32]|(RefSeq) phospholipase A(1) DAD1, chloroplastic (A)	8.454	8.019	
LOC106304780	K16818 phospholipase A1 [EC:3.1.1.32]|(RefSeq) phospholipase A(1) DAD1, chloroplastic (A)	3.539	2.836	
LOC106319091	K16818 phospholipase A1 [EC:3.1.1.32]|(RefSeq) phospholipase A(1) DAD1, chloroplastic (A)	1.288		
LOC106319092	K16818 phospholipase A1 [EC:3.1.1.32]|(RefSeq) phospholipase A(1) DAD1, chloroplastic (A)	2.360	2.589	
LOC106319835	K16818 phospholipase A1 [EC:3.1.1.32]|(RefSeq) phospholipase A(1) DAD1, chloroplastic (A)	5.064	5.207	
LOC106319836	K16818 phospholipase A1 [EC:3.1.1.32]|(RefSeq) phospholipase A(1) DAD1, chloroplastic (A)	3.195	2.749	
LOC106328260	K16818 phospholipase A1 [EC:3.1.1.32]|(RefSeq) phospholipase A(1) DAD1, chloroplastic (A)	8.278	8.389	
LOC106339443	K16818 phospholipase A1 [EC:3.1.1.32]|(RefSeq) phospholipase A(1) DAD1, chloroplastic (A)	1.116	1.087	
LOC106342041	K16818 phospholipase A1 [EC:3.1.1.32]|(RefSeq) phospholipase A(1) DAD1, chloroplastic (A)	−2.115	−1.209	
LOC106345019	K16818 phospholipase A1 [EC:3.1.1.32]|(RefSeq) phospholipase A(1) DAD1, chloroplastic (A)	5.876	7.322	1.475
LOC106320614	K14674 TAG lipase/steryl ester hydrolase/phospholipase A2/LPA acyltransferase [EC:3.1.1.3 3.1.1.13 3.1.1.4 2.3.1.51]|(RefSeq) triacylglycerol lipase SDP1-like (A)	−1.848		
LOC106327147	K14674 TAG lipase/steryl ester hydrolase/phospholipase A2/LPA acyltransferase [EC:3.1.1.3 3.1.1.13 3.1.1.4 2.3.1.51]|(RefSeq) triacylglycerol lipase SDP1-like (A)	10.204	10.611	
novel.962	K14674 TAG lipase/steryl ester hydrolase/phospholipase A2/LPA acyltransferase [EC:3.1.1.3 3.1.1.13 3.1.1.4 2.3.1.51]|(RefSeq) triacylglycerol lipase SDP1-like (A)		3.111	
LOC106318006	K14674 TAG lipase/steryl ester hydrolase/phospholipase A2/LPA acyltransferase [EC:3.1.1.3 3.1.1.13 3.1.1.4 2.3.1.51]|(RefSeq) triacylglycerol lipase SDP1 (A)	2.986	3.250	
novel.4548	K14674 TAG lipase/steryl ester hydrolase/phospholipase A2/LPA acyltransferase [EC:3.1.1.3 3.1.1.13 3.1.1.4 2.3.1.51]|(RefSeq) triacylglycerol lipase SDP1 (A)	3.224	3.019	
LOC106319652	K01047 secretory phospholipase A2 [EC:3.1.1.4]|(RefSeq) phospholipase A2-alpha (A)	−1.656	−2.641	
LOC106337350	K05857 phosphatidylinositol phospholipase C, delta [EC:3.1.4.11]|(RefSeq) phosphoinositide phospholipase C 7-like (A)	3.186	3.277	
LOC106296332	K05857 phosphatidylinositol phospholipase C, delta [EC:3.1.4.11]|(RefSeq) phosphoinositide phospholipase C 6 (A)	−4.671	−6.423	
LOC106322487	K05857 phosphatidylinositol phospholipase C, delta [EC:3.1.4.11]|(RefSeq) phosphoinositide phospholipase C 4 (A)	−1.106	−1.682	
LOC106324915	K05857 phosphatidylinositol phospholipase C, delta [EC:3.1.4.11]|(RefSeq) phosphoinositide phospholipase C 2 (A)	−1.890	−1.653	
LOC106312862	K05857 phosphatidylinositol phospholipase C, delta [EC:3.1.4.11]|(RefSeq) phosphoinositide phospholipase C 1 isoform X1 (A)	1.477		
LOC106322918	K05857 phosphatidylinositol phospholipase C, delta [EC:3.1.4.11]|(RefSeq) phosphoinositide phospholipase C 1 isoform X1 (A)	4.458	2.892	−1.535
LOC106308215	K01114 phospholipase C [EC:3.1.4.3]|(RefSeq) non-specific phospholipase C6-like (A)	−3.310	−2.155	
LOC106315255	K01114 phospholipase C [EC:3.1.4.3]|(RefSeq) non-specific phospholipase C6 (A)	−1.383	−1.067	
LOC106293041	K01114 phospholipase C [EC:3.1.4.3]|(RefSeq) non-specific phospholipase C4 (A)	12.631	13.033	
LOC106337025	K01114 phospholipase C [EC:3.1.4.3]|(RefSeq) non-specific phospholipase C2 (A)	−4.289	−3.061	
LOC106295442	K01114 phospholipase C [EC:3.1.4.3]|(RefSeq) non-specific phospholipase C1 (A)	2.006	1.927	
LOC106322994	K01115 phospholipase D1/2 [EC:3.1.4.4]|(RefSeq) phospholipase D zeta (A)	1.273	2.186	
LOC106331279	K01115 phospholipase D1/2 [EC:3.1.4.4]|(RefSeq) phospholipase D p2 (A)	4.193	4.655	
LOC106295624	K01115 phospholipase D1/2 [EC:3.1.4.4]|(RefSeq) phospholipase D p1 isoform X1 (A)	1.955	3.367	1.439
LOC106325483	K01115 phospholipase D1/2 [EC:3.1.4.4]|(RefSeq) phospholipase D delta-like (A)	1.248	1.688	
LOC106294863	K01115 phospholipase D1/2 [EC:3.1.4.4]|(RefSeq) phospholipase D alpha 1 (A)	1.137	1.049	
LOC106302179	K01115 phospholipase D1/2 [EC:3.1.4.4]|(RefSeq) phospholipase D zeta isoform X1 (A)		5.230	2.158
novel.2369	K01115 phospholipase D1/2 [EC:3.1.4.4]|(RefSeq) phospholipase D delta-like (A)		−1.715	−1.601
novel.3393	K01115 phospholipase D1/2 [EC:3.1.4.4]|(RefSeq) phospholipase D delta--like (A)		3.424	
LOC106310131	K01115 phospholipase D1/2 [EC:3.1.4.4]|(RefSeq) phospholipase D delta (A)		1.300	
LOC106327129	K01115 phospholipase D1/2 [EC:3.1.4.4]|(RefSeq) phospholipase D beta 2 (A)		1.262	
LOC106318460	K01115 phospholipase D1/2 [EC:3.1.4.4]|(RefSeq) phospholipase D gamma 1 (A)			1.227
LOC106294909	K01115 phospholipase D1/2 [EC:3.1.4.4]|(RefSeq) phospholipase D beta 1 (A)			1.251
Lipoxygenases				
LOC106300336	K00454 lipoxygenase [EC:1.13.11.12]|(RefSeq) lipoxygenase 6, chloroplastic (A)	−1.193		
LOC106327768	K00454 lipoxygenase [EC:1.13.11.12]|(RefSeq) lipoxygenase 4, chloroplastic (A)	1.154	2.598	1.466
LOC106312730	K00454 lipoxygenase [EC:1.13.11.12]|(RefSeq) lipoxygenase 3, chloroplastic (A)	2.457	4.275	1.843
LOC106300136	K00454 lipoxygenase [EC:1.13.11.12]|(RefSeq) lipoxygenase 2, chloroplastic-like (A)	−4.832	−3.396	1.460
LOC106300607	K00454 lipoxygenase [EC:1.13.11.12]|(RefSeq) lipoxygenase 2, chloroplastic-like (A)	−4.983	−5.284	
LOC106320988	K00454 lipoxygenase [EC:1.13.11.12]|(RefSeq) lipoxygenase 2, chloroplastic-like (A)	−4.226	−6.821	
LOC106321232	K00454 lipoxygenase [EC:1.13.11.12]|(RefSeq) lipoxygenase 2, chloroplastic-like (A)	−6.015	−3.496	2.553
LOC106321533	K00454 lipoxygenase [EC:1.13.11.12]|(RefSeq) lipoxygenase 2, chloroplastic-like (A)	−7.140	−4.851	
LOC106327154	K00454 lipoxygenase [EC:1.13.11.12]|(RefSeq) lipoxygenase 2, chloroplastic-like (A)	−7.181	−7.124	
novel.4542	K00454 lipoxygenase [EC:1.13.11.12]|(RefSeq) lipoxygenase 2, chloroplastic-like (A)	−5.196	−2.852	
novel.4597	K00454 lipoxygenase [EC:1.13.11.12]|(RefSeq) lipoxygenase 2, chloroplastic-like (A)	−7.214	−2.423	4.829
novel.4661	K00454 lipoxygenase [EC:1.13.11.12]|(RefSeq) lipoxygenase 2, chloroplastic-like (A)	−5.834	−7.073	
LOC106311934	K00454 lipoxygenase [EC:1.13.11.12]|(RefSeq) lipoxygenase 3, chloroplastic (A)			1.458

**Table 3:** Differentially expressed Mg-dechelatase (SGR), pheophorbide a oxygenase (PAO), red chlorophyll catabolite reductase (RCCR), and pheophorbidase (PPD) genes identified in CK5 vs. CK0, MT5 vs. CK0, and/or MT5 vs. CK5.

Accession No.	KEGG	log_2_ (FC)		
		Group A	Group B	Group C
LOC106332354	K22013 magnesium dechelatase [EC:4.99.1.10]|(RefSeq) protein STAY-GREEN 2, chloroplastic-like isoform X1 (A)	7.819	8.161	
LOC106311628	K22013 magnesium dechelatase [EC:4.99.1.10]|(RefSeq) protein STAY-GREEN 1, chloroplastic-like isoform X1 (A)	2.534	2.620	
LOC106306852	K22013 magnesium dechelatase [EC:4.99.1.10]|(RefSeq) protein STAY-GREEN 1, chloroplastic (A)	4.095	4.475	
LOC106296745	K22013 magnesium dechelatase [EC:4.99.1.10]|(RefSeq) protein STAY-GREEN LIKE, chloroplastic (A)		−1.333	
LOC106314152	K13071 pheophorbide a oxygenase [EC:1.14.15.17]|(RefSeq) pheophorbide a oxygenase, chloroplastic (A)	1.354	1.547	
LOC106314162	K13071 pheophorbide a oxygenase [EC:1.14.15.17]|(RefSeq) pheophorbide a oxygenase, chloroplastic (A)		−1.216	
LOC106335512	K13071 pheophorbide a oxygenase [EC:1.14.15.17]|(RefSeq) pheophorbide a oxygenase, chloroplastic (A)		1.042	
LOC106325027	K13545 red chlorophyll catabolite reductase [EC:1.3.7.12]|(RefSeq) red chlorophyll catabolite reductase, chloroplastic (A)	1.111		
LOC106293153	K13544 pheophorbidase [EC:3.1.1.82]|(RefSeq) pheophorbidase-like (A)	3.565	1.714	−1.818
LOC106304188	K13544 pheophorbidase [EC:3.1.1.82]|(RefSeq) pheophorbidase-like (A)	1.005		−1.712
LOC106311503	K13544 pheophorbidase [EC:3.1.1.82]|(RefSeq) pheophorbidase-like (A)	−2.709	−2.872	
LOC106324413	K13544 pheophorbidase [EC:3.1.1.82]|(RefSeq) pheophorbidase-like (A)	2.273		
LOC106325575	K13544 pheophorbidase [EC:3.1.1.82]|(RefSeq) pheophorbidase-like (A)	−1.693	−2.428	
LOC106327219	K13544 pheophorbidase [EC:3.1.1.82]|(RefSeq) pheophorbidase-like (A)	−1.331		
LOC106292376	K13544 pheophorbidase [EC:3.1.1.82]|(RefSeq) pheophorbidase (A)	5.330	4.156	−1.129
LOC106299990	K13544 pheophorbidase [EC:3.1.1.82]|(RefSeq) pheophorbidase (A)	1.322		
LOC106342417	K13544 pheophorbidase [EC:3.1.1.82]|(RefSeq) pheophorbidase (A)	1.536		
novel.873	K13544 pheophorbidase [EC:3.1.1.82]|(RefSeq) pheophorbidase (A)	2.179	2.396	
LOC106303906	K13544 pheophorbidase [EC:3.1.1.82]|(RefSeq) pheophorbidase-like (A)		2.612	2.227
LOC106312561	K13544 pheophorbidase [EC:3.1.1.82]|(RefSeq) pheophorbidase-like (A)		−1.883	−1.336

**Table 4:** Differentially expressed peroxisomal citrate synthase and peroxin (peroxisomal membrane protein) genes identified in CK5 vs. CK0, MT5 vs. CK0, and/or MT5 vs. CK5.

Accession No.	KEGG	log_2_ (FC)		
		Group A	Group B	Group C
LOC106339255	K13347 peroxisomal membrane protein 2|(RefSeq) peroxisomal membrane protein PMP22 (A)	1.536	2.542	1.029
LOC106293462	K13345 peroxin-12|(RefSeq) peroxisome biogenesis protein 12 (A)	−1.568		
LOC106334624	K13344 peroxin-13|(RefSeq) peroxisomal membrane protein 13-like isoform X1 (A)	1.455	1.603	
LOC106302538	K13344 peroxin-13|(RefSeq) peroxisomal membrane protein 13-like (A)	3.091		
LOC106295513	K13344 peroxin-13|(RefSeq) peroxisomal membrane protein 13 (A)	1.822	1.044	
LOC106313303	K13344 peroxin-13|(RefSeq) peroxisomal membrane protein 13 (A)	−3.408	−8.604	−5.152
LOC106321286	K13344 peroxin-13|(RefSeq) peroxisomal membrane protein 13 (A)	−1.974		
LOC106319419	K13343 peroxin-14|(RefSeq) peroxisomal membrane protein PEX14-like (A)	1.511	1.357	
LOC106295293	K13339 peroxin-6|(RefSeq) LOW QUALITY PROTEIN: peroxisome biogenesis protein 6-like (A)	−2.188	−3.004	
LOC106341678	K13336 peroxin-3|(RefSeq) peroxisome biogenesis protein 3-1-like (A)	−1.125		
LOC106337474	K13335 peroxin-16|(RefSeq) peroxisome biogenesis protein 16 (A)	−1.216	−2.226	
LOC106307741	K13346 peroxin-10|(RefSeq) peroxisome biogenesis factor 10 isoform X1 (A)		1.023	1.321
LOC106307054	K13345 peroxin-12|(RefSeq) peroxisome biogenesis protein 12 (A)		2.143	1.474
LOC106335680	K13342 peroxin-5|(RefSeq) peroxisome biogenesis protein 5-like (A)		1.153	
LOC106311817	K13339 peroxin-6|(RefSeq) peroxisome biogenesis protein 6 (A)		1.218	
LOC106342475	K01647 citrate synthase [EC:2.3.3.1]|(RefSeq) citrate synthase 3, peroxisomal (A)	2.598	2.694	
LOC106301011	K01647 citrate synthase [EC:2.3.3.1]|(RefSeq) citrate synthase 2, peroxisomal-like (A)	2.912	3.199	
LOC106339817	K01647 citrate synthase [EC:2.3.3.1]|(RefSeq) citrate synthase 2, peroxisomal-like (A)	−4.024	−2.341	
LOC106311978	K01647 citrate synthase [EC:2.3.3.1]|(RefSeq) citrate synthase 2, peroxisomal (A)	4.139	3.753	
LOC106311979	K01647 citrate synthase [EC:2.3.3.1]|(RefSeq) citrate synthase 2, peroxisomal-like (A)		9.023	1.253

## Data Availability

Data will be made available on request. RNA-Seq data were deposited in the NCBI database with SRA accession number PRJNA1347445 (https://www.ncbi.nlm.nih.gov/bioproject/PRJNA1347445, accessed on 22 October 2025).

## Supplementary Materials

The following supporting information can be downloaded at: https://www.mdpi.com/article/10.3390/plants15060870/s1, Figure S1: RT-qPCR confirmation of 10 DEGs identified in CK0, CK5, MT5; Figure S2: Score scatter plots of OPLS-DA model and permutation test of OPLS-DA model for CK5 vs. CK0, MT5 vs. CK0, and MT5 vs. CK5; Table S1: Specific primer pairs used for RT-qPCR analysis; Table S2: Summary of the RNA-Seq data collected from broccoli florets in the control (CK) and static magnetic field treatment (MT) on 0 and 5d; Table S3: Summary of clean reads and genes mapped to the reference genome from broccoli florets in the control (CK) and static magnetic field treatment (MT) at 0 and 5d; Table S4: List of DEGs identified in CK5 vs. CK0; Table S5: List of DEGs identified in MT5 vs. CK0; Table S6: List of DEGs identified in MT5 vs. CK5; Table S7: List of enriched Go terms for DEGs in CK5 vs. CK0; Table S8: List of enriched Go terms for DEGs in MT5 vs. CK0; Table S9: List of enriched Go terms for DEGs in MT5 vs. CK5; Table S10: List of enriched KEGG pathways for DEGs in CK5 vs. CK0; Table S11: List of enriched KEGG pathways for DEGs in MT5 vs. CK0.; Table S12: List of enriched KEGG pathways for DEGs in MT5 vs. CK5; Table S13: List of differentially expressed metabolites (DAMs) identified in CK5 vs. CK0; Table S14: List of differentially expressed metabolites (DAMs) identified in MT5 vs. CK0; Table S15: List of differentially expressed metabolites (DAMs) identified in MT5 vs. CK5; Table S16: Statistical analysis of the DAMs identified in CK5 vs. CK0, MT5 vs. CK0 and MT5 vs. CK5.; Table S17: List of differentially abundant free fatty acids (FFAs) identified in CK5 vs. CK0, MT5 vs. CK0, and MT5 vs. CK5; Table S18: List of enriched KEGG pathways for DAMs in CK5 vs. CK0; Table S19: List of enriched KEGG pathways for DAMs in MT5 vs. CK0; Table S20: List of enriched KEGG pathways for DAMs in MT5 vs. CK5.

## Abbreviations

The following abbreviations are used in this manuscript:

1-MCP	1-methylcyclopropene
AAs	amino acids
BP	biological process
CC	cellular component
CoA	acetyl-coenzyme A
DAG	diacylglycerol
FFAs	free fatty acids
HCA	hierarchical cluster analysis
LOX	lipoxygenase
LPLs	lysophospholipids
LPCs	lysophosphatidylcholines
LPEs	lysophosphatidylethanolamines
MDA	malondialdehyde
MeJA	methyl jasmonate
MF	magnetic field
MT	SMF treatment
OAs	organic acids
PA	phosphatidic acid
PAO	pheophorbide a oxygenase
PBDs	peroxisome biogenesis disorders
PC	phosphatidylcholine
PCCs	Pearson’s correlation coefficients
PL	phospholipid
PLAs	phospholipase As
PLD	phospholipase D
PPD	pheophorbidase
RCCR	red chlorophyll catabolite reductase
SGR	magnesium-dechelatase
SMF	static magnetic field
SNP	sodium nitroprusside
TBA	thiobarbituric acid
TCA	trichloroacetic acid
