# Supplementary material for: Integrated Multi-Omics Analysis Reveals Lipid Metabolism-Mediated Preservation of Postharvest Broccoli Yellowing by Static Magnetic Field
Source: Plants (Basel). 2026 Mar 11;15(6):870. doi: 10.3390/plants15060870 (PMC13029757; doi:10.3390/plants15060870)
Supplement: Supplementary file 1 [file plants-15-00870-s001.zip › Figures S1-S2.pdf]

Supplementary Figures S1-S2

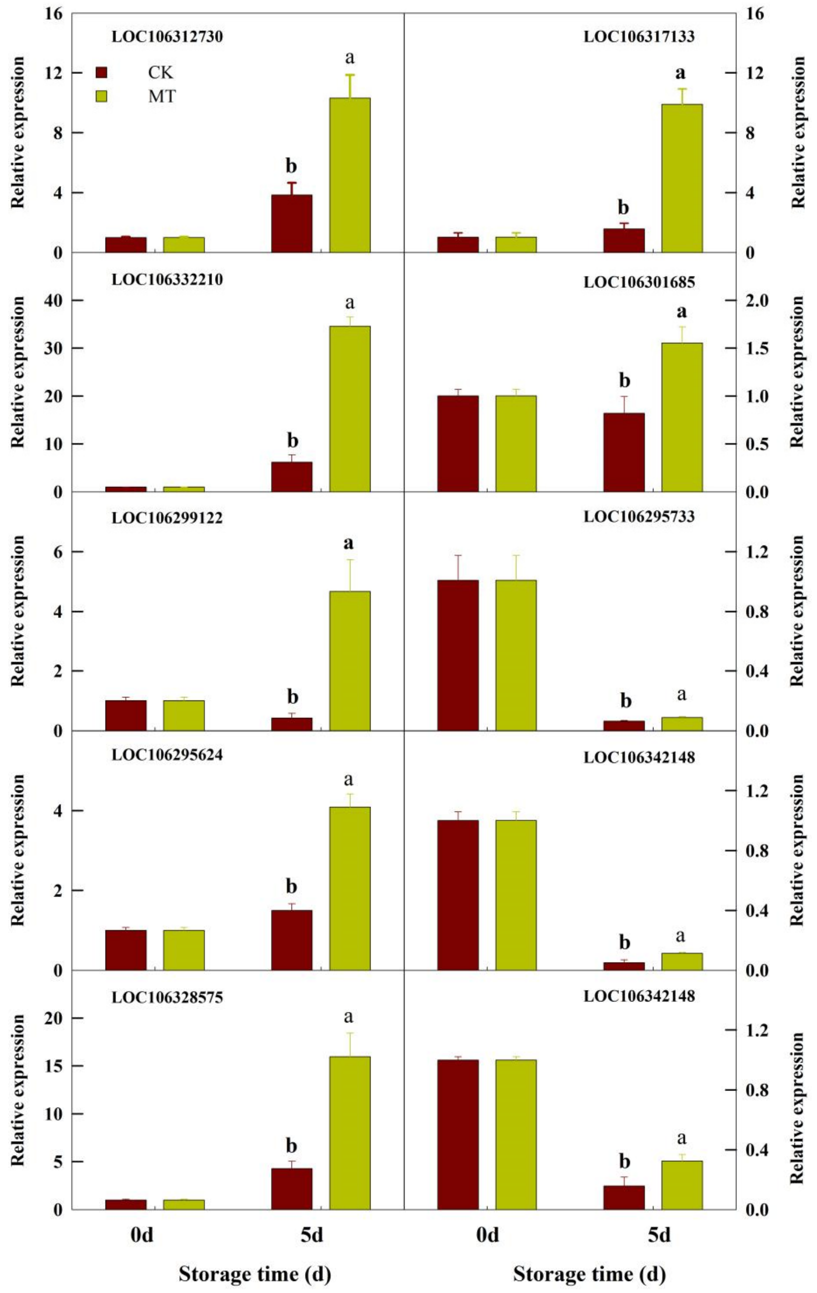

Figure S1. RT-qPCR confirmation of 10 DEGs identified in CK0, CK5, MT5. Bars represent means ± SE of 3 technical and 3 biological replicates. For the same genes, different letters above the bars indicate a significant difference at $p < 0.05$.

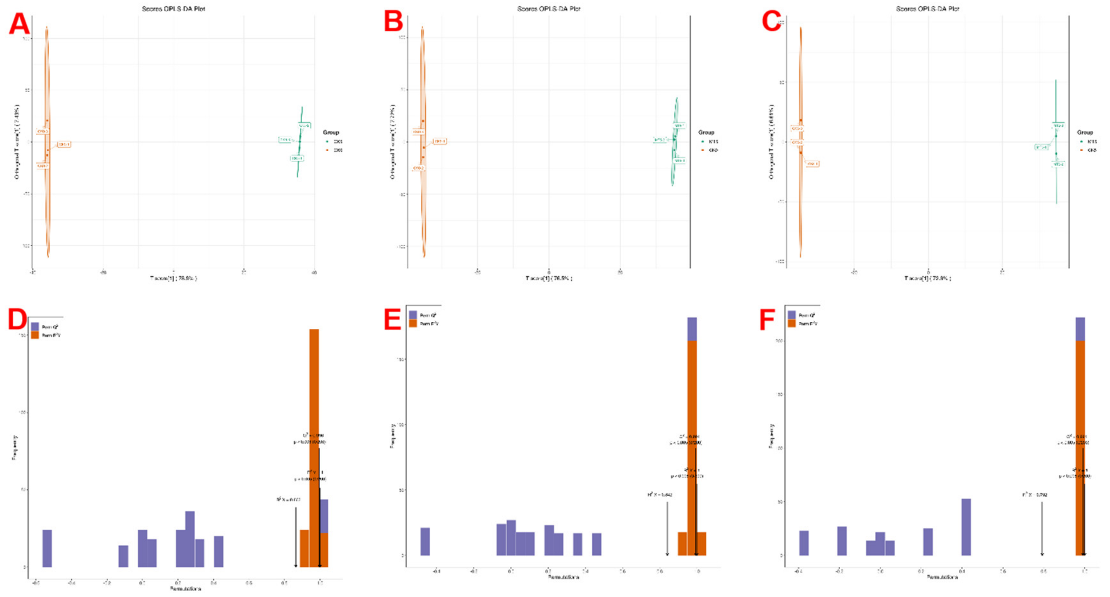

Figure S2. Score scatter plots of OPLS-DA model (A-C) and permutation test of OPLS-DA model (D-F) for CK5 vs CK0 (A and D), MT5 vs CK0 (B and E), and MT5 vs CK5 (C and F).
