# Supplementary material for: Integrated Multi-Omics Analysis Reveals Lipid Metabolism-Mediated Preservation of Postharvest Broccoli Yellowing by Static Magnetic Field
Source: Plants (Basel). 2026 Mar 11;15(6):870. doi: 10.3390/plants15060870 (PMC13029757; doi:10.3390/plants15060870)
Supplement: Supplementary file 1 [file plants-15-00870-s001.zip › Tables S1-S3.pdf]

Supplementary Tables S1-S3

Table S1. Specific primer pairs used for RT-qPCR analysis

ID	Description	Forward primers (5′→3′)	Reverse primers (5′→3′)
LOC106312730	Lipoxygenase 3, chloroplastic	TGGCTACGAGATGACGAGTT	GAAGTGAGAGCGGAGTGTTG
LOC106332210	Fatty alcohol:caffeoyl-CoA acyltransferase	AATGTGGAGGATTCGTGTTAGG	CAAGAAAGGTGGGACTGAGAG
LOC106299122	Cytochrome P450 704B1; Long-chain fatty acid omega-hydroxylase	TTCGTTTCATAGACCCTCTTTGG	TGCTTCATCTTAGTGTTGTTGGA
LOC106295624	Phospholipase D zeta 1	GCAGCATCAGTTAGAGCCATAA	CGGAAAGTTTATCATACGCCCTA
LOC106328575	Beta-galactosidase 6	ATTCTGGCTATGGTTCTTGTTCTC	GTGTGCTGCGAGGATAATGAA
LOC106317133	Glycerol-3-phosphate 2-O-acyltransferase 6	GCTTCGTGCGTTGATCTTACT	ACTCTTTCTCGTAGACTCTTGCTA
LOC106301685	Diacylglycerol O-acyltransferase 1	GGAGAATGTGGAATATGCCTGTT	TTGAAGAGACGACAAGGAACTG
LOC106295733	3-ketoacyl-CoA synthase 2	ACCTCTCGTCTTGCCTATGT	TCATCCAACACTGCTCTACCT
LOC106342148	Long chain acyl-CoA synthetase 1	ATGCTTGTTGCGGTGATTGT	TTCTTCTCTGCCGTGGACTT
LOC106327484	Very-long-chain aldehyde decarbonylase CER3	CCTTAGAGTTCGTGTGGTTCAT	TAGAAGTGGCTCCTGTCAAGA
Actin		CCAGAGGTCTTGTTCCAGCCATC	GTTCCACCACTGAGCACAATGTTAC

The sequences of Forward and Reverse primers for these DEGs were designed using Primer Premier version 5.0 (Premier Biosoft International, CA, USA).

Table S2. Summary of the RNA-Seq data collected from broccoli florets in the control (CK) and static magnetic field treatment (MT) on 0 and 5d.

Sample	Raw Reads	Clean Reads	Clean Base(G)	Error Rate(%)	Q20(%)	Q30(%)	GC Content(%)
CK0-1	47353148	46719262	7.01	0.01	97.96	94.03	48.60
CK0-2	47276486	46571000	6.99	0.02	96.80	90.82	49.58
CK0-3	44417404	43829178	6.57	0.02	96.84	90.90	49.69
CK5-1	42992680	42172586	6.33	0.02	96.59	90.29	49.97
CK5-2	51711976	50860766	7.63	0.02	96.93	91.19	49.75
CK5-3	45224392	44478478	6.67	0.02	96.75	90.70	50.35
MT5-1	49261762	48612572	7.29	0.01	97.85	93.67	48.69
MT5-2	48713880	48016054	7.20	0.01	97.39	92.43	49.47
MT5-3	45839294	45170724	6.78	0.02	96.64	90.39	49.85

Table S3. Summary of clean reads and genes mapped to the reference genome from broccoli florets in the control(CK) and static magnetic field treatment (MT) on 0 and 5d.

Sample	Total Reads	Reads mapped	Unique mapped	Multi mapped	Read1 mapped	Read2 mapped	'+' mapped	'-' mapped
CK0-1	46719262	41011667(87.78%)	39910047(85.43%)	1101620(2.36%)	20162728(43.16%)	19747319(42.27%)	19943125(42.69%)	19966922(42.74%)
CK0-2	46571000	40572709(87.12%)	39431882(84.67%)	1140827(2.45%)	19843987(42.61%)	19587895(42.06%)	19670648(42.24%)	19761234(42.43%)
CK0-3	43829178	38202115(87.16%)	37149370(84.76%)	1052745(2.40%)	18678180(42.62%)	18471190(42.14%)	18532257(42.28%)	18617113(42.48%)
CK5-1	42172586	34034323(80.70%)	32955184(78.14%)	1079139(2.56%)	16613612(39.39%)	16341572(38.75%)	16463545(39.04%)	16491639(39.11%)
CK5-2	50860766	41364529(81.33%)	39999556(78.65%)	1364973(2.68%)	20150586(39.62%)	19848970(39.03%)	19983976(39.29%)	20015580(39.35%)
CK5-3	44478478	35700697(80.27%)	34542039(77.66%)	1158658(2.60%)	17386181(39.09%)	17155858(38.57%)	17245079(38.77%)	17296960(38.89%)
MT5-1	48612572	43236404(88.94%)	42021992(86.44%)	1214412(2.50%)	21235362(43.68%)	20786630(42.76%)	21004360(43.21%)	21017632(43.23%)
MT5-2	48016054	42137609(87.76%)	41011009(85.41%)	1126600(2.35%)	20825754(43.37%)	20185255(42.04%)	20511681(42.72%)	20499328(42.69%)
MT5-3	45170724	39543371(87.54%)	38384409(84.98%)	1158962(2.57%)	19336053(42.81%)	19048356(42.17%)	19173292(42.45%)	19211117(42.53%)
